# In situ mechanostimulation of biohybrid millirobots for enhanced cell functionality and delivery

**DOI:** 10.1126/sciadv.adx9616

**Published:** 2026-01-02

**Authors:** Jianhua Zhang, Xianqiang Bao, Zhou Zhu, Rongjing Zhang, Chunxiang Wang, Mingtong Li, Kaichen Xu, Yong He, Dietmar W. Hutmacher, Ziyu Ren, Metin Sitti

**Affiliations:** ^1^ State Key Laboratory of Fluid Power and Mechatronic Systems & Liangzhu Laboratory, School of Mechanical Engineering, Zhejiang University, Hangzhou 310027, China.; ^2^ Physical Intelligence Department, Max Planck Institute for Intelligent Systems, Stuttgart 70569, Germany.; ^3^ Max Planck Queensland Center (MPQC) for the Materials Science of Extracellular Matrices, Queensland University of Technology, Brisbane, QLD 4000, Australia.; ^4^ Jiangsu Provincial Joint International Research Laboratory of Medical Information Processing, School of Computer Science and Engineering, Southeast University and Key Laboratory of New Generation Artificial Intelligence Technology and Its Interdisciplinary Applications (Southeast University), Ministry of Education, Nanjing 211189, China.; ^5^ State Key Laboratory of Oral Diseases, National Clinical Research Center for Oral Diseases, West China Hospital of Stomatology, Sichuan University, Chengdu 610041, China.; ^6^ School of Mechanical Engineering and Automation, Beihang University, Beijing 100191, China.; ^7^ Australian Research Council Training Centre for Cell and Tissue Engineering Technologies, Queensland University of Technology, 1 George Street, Brisbane, QLD 4000, Australia.; ^8^ School of Medicine and College of Engineering, Koç University, Istanbul 34450, Turkey.

## Abstract

This study proposes a perforated, soft millirobot with dual functions: in situ mechanostimulation to enhance cell functionality and local cell delivery. Following protein modification and silica coating, the soft millirobots exhibit excellent biocompatibility, promoting cell adhesion and tissue ingrowth within their perforated architectures under both in vitro and in vivo conditions. They can apply in situ mechanostimulation to various cellular morphologies, including two-dimensional (2D) cell sheets, 3D cell–laden hydrogels, and ex vivo tissue models. The mechanical stimulation improves the functionality of muscle cells by enhancing cellular orientation, myotube contraction, and myocyte differentiation. In parallel, we develop an integrated robotic platform combining magnetic actuation with ultrasound imaging. It demonstrates the proof of principle that delivers 2D cell-sheet and 3D cell–laden biohybrid millirobots to narrow regions in an ex vivo pig liver model. This work expands the potential applications of soft millirobots in mechanobiology studies and future cell-based therapies.

## Teaser

Magnetic biohybrid millirobots enable in situ mechanostimulation and targeted cell delivery for tissue regeneration.

## INTRODUCTION

Cell-based therapeutics has emerged with the potential to treat many currently intractable diseases through uniquely powerful modes of action (*1*–*3*). The most convenient and direct method for cell delivery is the injection of a cell suspension directly into diseased tissues. This straightforward approach can be effectively used in conditions, such as osteoarthritis, where cells can be easily administered to the affected tissues via intra-articular or intratendinous injection (*4*). However, in cases involving substantial structural defects or internal tissue and organ delivery, the injections may lead to side effects, complications, and low cell concentration at the lesion site due to biological fluid flow–related cell loss (*5*). The efficacy of cell-based therapy depends on the precise delivery of viable cells to target sites, where cells can exert therapeutic effects through migration, proliferation, and differentiation, thereby facilitating tissue and organ repair.

To improve the low targeting efficiency of current cell-based therapies, some researchers have proposed the use of magnetically actuated microrobots for targeted cell delivery (*6*–*8*). The microrobots are assembled by seeding cells on a porous magnetic microstructure fabricated by two-photon polymerization three-dimensional (3D) microprinting (*2*, *9*, *10*) or forming magnetic spheroids through a coculture process of cells and magnetic iron oxide particles (*11*, *12*). To achieve time-efficient delivery of cell-based microrobots, some research groups have developed endoscopy-assisted magnetic actuation for rapid precision delivery to tiny regions (*11*). However, despite these promising results, most microrobots lack a cell-permissive environment, which provides biological and physical signals, necessary for cellular functions (*13*). The viability of cells within magnetic spheroids is notably influenced by the concentration of magnetic particles, and the coculture process may alter cellular functions. In addition, the cell-loading efficiency of microrobots is low. For example, clinical trials require 1 × 10^6^ to 2.5 × 10^6^ cells per cm^2^ at a lesion site (*2*), a target that is challenging to achieve with current cell-transport microrobots. Therefore, achieving delivery efficiency that meets therapeutic requirements remains a significant challenge in cell therapy.

Recent years have seen remarkable advancements in soft millirobots that operate using different locomotion modes, including rolling (*14*), climbing (*15*), crawling (*16*), and swimming (*17*–*19*), to navigate and function inside unstructured, confined, and varying biological environments. These magnetic soft millirobots have shown potential applications in medicine, such as targeted drug delivery (*20*), in situ sensing (*21*), endoscopy (*22*), and minimally invasive surgery (*23*), where precise movement within the human body is essential. Compared to microrobots for cell-based therapy, biohybrid millirobots offer potential advantages, such as easier magnetic actuation control, greater cell-loading efficiency, various locomotion modes, and enhanced medical imaging–guided navigation. However, several critical requirements for biohybrid millirobots should be further considered to improve the effectiveness of cell therapy. (i) Biocompatibility and cell integration: Current soft millirobots are mostly composed of nondegradable nonmagnetic and magnetic materials whose clinical safety has not been fully verified. Meanwhile, previous reports also indicate that cells seeded on the surface of soft patches may integrate slowly with the host tissue (*24*). (ii) Functionality: Some research has shown that cells cultured on 2D substrates rapidly lose their function and morphology (*25*). Similarly, cells integrated into biohybrid millirobots may lose their functions because of the absence of a biological and physical microenvironment similar to that of the in vivo environment.

Recent developments in soft robotics and cell-based therapeutics have opened possibilities for minimally invasive tissue repair and regeneration. However, current magnetic or electric robots are primarily designed for simple cell transportation and lack the ability to provide a biologically relevant mechanical microenvironment that supports cell functionality (*2*). Existing stimulation systems, including motor-driven or dielectric actuators, are generally bulky and restricted to global loading on 2D cultures, making localized and dynamic regulation of cell behavior challenging (*26*). Inspired by the self-strengthening of skeletal muscles by physical exercise, where the contraction-relaxation of biceps leads to the destruction and reconstruction of muscle fibrils, a similar approach can be applied to biohybrid millirobots to mimic the native mechanical environment. In our previous study, we demonstrated that long-term mechanical stimulation in a compression bioreactor is essential for enhancing osteocyte differentiation and the formation of 3D osteocyte bone organoids from stem cells (*27*). Localized mechanical stimulation at the site of small muscle injuries via cyclic contraction-relaxation training has been shown to improve functional recovery in mice by rapidly clearing neutrophils that could impede myogenesis (*28*). When cells are integrated into these millirobots, applying mechanical stimulation becomes a promising approach to improve cellular functionality. In addition, a magnetic field, which is transparent and relatively safe for biological tissues, serves as a powerful tool for the remote actuation and wireless control of magnetic soft millirobots, enabling the application of diverse cyclic mechanical strains and stimulations on cells.

Here, we introduce a magnetic soft millirobot that integrates dual functions: in situ mechanostimulation and localized cell delivery, within a single biocompatible platform. The perforated and fibronectin-modified structure provides a highly cell-permissive surface for cell adhesion and proliferation, while wireless magnetic actuation enables reversible contraction-relaxation transformations that mimic natural muscle movement. This design achieves localized, cyclic mechanical conditioning of cells and precise, ultrasound (US)–guided delivery into confined tissue regions, offering a previously unknown route to combine mechanobiological regulation with minimally invasive therapeutic delivery.

## RESULTS

### Fabrication and actuation of perforated sheet-shaped millirobots

**Fig. 1.**
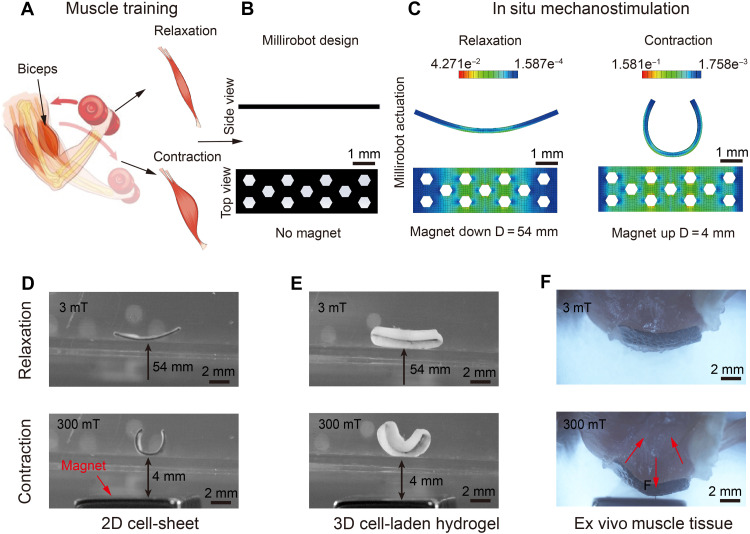
In situ mechanostimulation of soft perforated millirobots with different cellular morphologies under magnetic actuation. (**A**) Muscle training process, (**B**) millirobot design, (**C**) FE analysis, and (**D** to **F**) contraction and relaxation training of soft perforated millirobots on 2D cell-sheet, 3D cell-laden hydrogel, and ex vivo mouse muscle model.

Drawing inspiration from the muscle training process, which involves the contraction and relaxation of the biceps (Fig. 1A), we propose the design of a perforated, sheet-shaped soft millirobot to mimic this process, applying in situ mechanostimulation to cells under magnetic actuation. The selected base materials for the fabrication of millirobots are ferromagnetic neodymium-iron-boron (NdFeB) microparticles and soft elastomer polydimethylsiloxane (PDMS, Sylgard 184). To prevent corrosion and direct contact with the tissue, the embedded NdFeB particles are coated with a thin silica shell (NdFeB@SiO_2_), as reported in previous study (*29*). The biocompatibility of NdFeB@SiO_2_ is improved compared to bare NdFeB particles. As shown in fig. S1, after coculturing with 3T3 fibroblast cells, the cell viability of 3T3 in NdFeB@SiO_2_ groups is significantly higher than in NdFeB groups on days 1 and 7. Cell viability decreases with increasing particle concentration but remains above 80% when the concentration is below 1 mg/ml on day 7. The structural stability and anticorrosion ability of magnetic NdFeB@SiO_2_ particles before and after 21 days of in vitro culture are shown in figs. S2 and S3.

The fabrication process of the sheet-shaped millirobots, previously developed in our earlier studies (*30*, *31*), has been adapted to incorporate NdFeB@SiO_2_ particles instead of NdFeB particles. The millirobots developed in this study have a specific width (2 mm), thickness (0.2 mm), and length (6.4 mm), which ensures that the length-to-thickness ratio is at least 11:1 to achieve large deformations. To enhance the integration capability of cells and hydrogels with the fabricated soft millirobots and facilitate the migration of cells to tissues, patterned structures (hexagon, square, and triangle) are cut to create perforated millirobots using laser micromachining. Each unit has a length of 0.5 mm, with a 1-mm spacing between adjacent units. The perforated millirobots are illustrated in Fig. 1B and fig. S4.

After magnetization under 1.8-T uniform magnetic field with an initial phase shift of 0°, the perforated sheet-shaped millirobots have a harmonic magnetization profile with actuation versatility in producing multimodal soft-bodied locomotion (*14*, *30*). Magnetic hysteresis characteristics of millirobot materials (fig. S5), which is obtained by a vibrating sample magnetometer (VSM) under varying magnetic fields. The magnetization magnitude is 80.95 ± 5.07 kA/m. The NdFeB@SiO_2_ microparticles tend to align their magnetization directions with the external magnetic field, generating torques that deform the soft elastomeric millirobot body (*32*, *33*). Reversible contraction-relaxation transformation training can be repeated on demand by magnetic actuation. To enable this, we have engineered a device capable of reciprocating motion. This apparatus integrates a magnetic platform, a bipolar stepper motor, and a crank arm linkage, as depicted in fig. S6 and movie S1. The magnetic platform is capable of cyclic vertical movements within a range of 4 to 54 mm, driven by the motor. The experimental magnetic field strength applied to the millirobots ranges from 2 to 300 mT. By regulating the duty cycle of electrical pulses applied to the motor, the frequency of millirobot actuation, induced by the upward and downward cycles of the magnetic platform, can be precisely controlled at 1 Hz. The millirobot demonstrates cyclical deformation movements of contraction and relaxation modes in response to cyclic vertical movements of the magnet under the millirobot.

To simulate the deformation of perforated millirobot, the neo-Hookean model is used to model the strain-stress relationship and predict the transformation under magnetic fields in the commercial finite-element (FE) analysis software ABAQUS (*33*). The simulation results predict the strain change in contraction-relaxation deformations on the millirobot surface (Fig. 1C), which induces in situ mechanostimulation on cells integrated to our biohybrid millirobots. On the basis of the FE stimulation and experimental designs, three different biohybrid millirobots have been introduced to induce in situ mechanostimulation on cells in different cellular morphologies from 2D cell-sheet, 3D cell-laden hydrogels, and ex vivo tissue models (Fig. 1, D to F). To further quantify the magnetic actuation performance, we estimated the magnetic torque required to achieve the observed deformation of the soft millirobots (*14*). The analysis shows that a torque of approximately 1.98 × 10^−5^ N/m is required to produce the maximum deformation observed in millirobots. Meanwhile, the millirobot’s actuation generates velocity gradients and vortex flow waves that substantially enhance local fluid transport and mixing, potentially contributing to improved microcirculation (*34*). This effect is supported by dye-tracing (fig. S7) and particle image velocimetry (fig. S8) experiments.

### Effects of 2D biohybrid millirobot actuation on cell growth

**Fig. 2.**
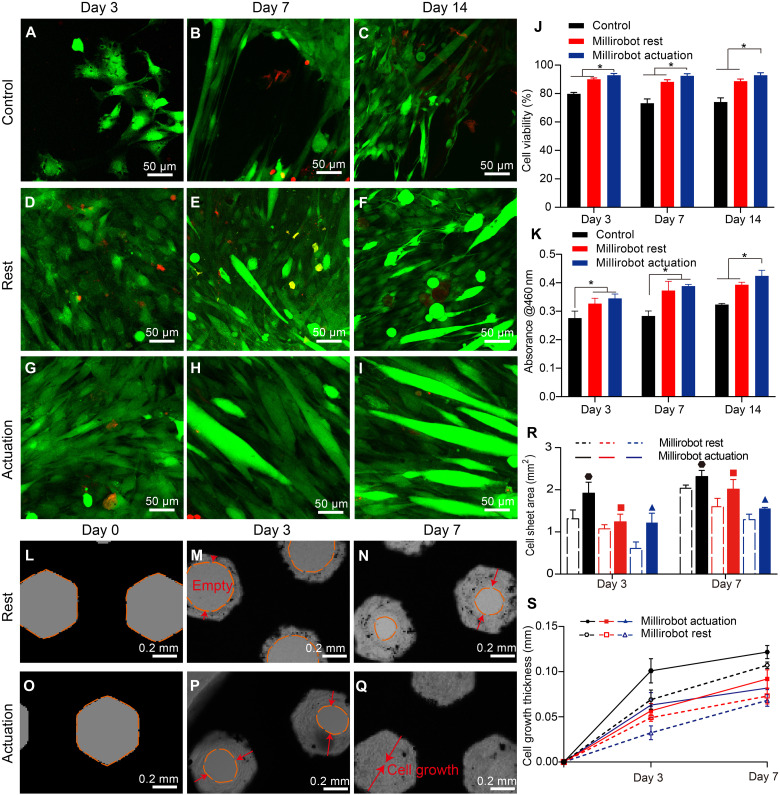
Biocompatibility of the 2D cell-sheet soft perforated millirobot and effects of in situ mechanostimulation on the C2C12 cell growth. (**A** to **I**) Live-dead staining images of control (A to C), millirobot rest (D to F), and millirobot actuation (G to I) on day 3 (A, D, and G), day 7 (B, E, and H), and day 14 (C, F, and I); (**J**) cell viability, (**K**) cell proliferation, *P < 0.05. (**L** to **Q**) Bright-field images showing cell-sheet growth in the millirobot with hexagonal pore structures under millirobot rest (L to N) and actuation (O to Q) conditions on day 0 (L and O), day 3 (M and P), and day 7 (N and Q). (**R** and **S**) Analysis of cell-sheet area and cell growth thickness with different pore structures in millirobot rest and actuation conditions.

To support cell attachment, 2D-perforated millirobots are modified by covalently binding fibronectin to their surface (*35*). The formation of 2D biohybrid millirobots is coculturing cells with 2D-perforated millirobots on a nonadhesive surface of the well plate. We are investigating the influence of in situ mechanostimulation on cell growth with different cell types, including human mesenchymal stem cells (hMSCs), National Institutes of Health (NIH) mouse fibroblast cells (3T3), and mouse myoblast cells (C2C12). As shown in Fig. 2 and figs. S9 and S10, fibronectin modification and mechanical stimulation significantly improve cell viability and cell proliferation. With fibronectin modification, cell spreading behavior and cell area of C2C12, hMSCs, and 3T3 cells are improved on days 3, 7, and 14, which is similar to previous reports (*35*, *36*). We attribute this positive effect to the surface activation effect of the elastomer and the fibronectin concentration, which are coupled with the molecular conformations (*37*). In addition, fibronectin molecules may help prevent corrosion in the exposure zones of sheet-shaped millirobots with various patterned structures.

Cells responded to in situ mechanical stimulation induced by millirobot actuation in vitro at both the cellular (viability and morphology) and molecular (proliferation) levels. C2C12 cells were subjected to different frequencies and durations, and proliferation was measured on days 1, 3, and 6. Cells stimulated at 1 Hz exhibited the highest proliferation compared with 0.5 Hz, 1.5 Hz, and control (fig. S11). Proliferation decreased at 1.5 Hz, likely because higher-frequency stimulation imposes excessive mechanical stress that can damage cells and inhibit growth. At 1 Hz, a duration of 1 hour/day further enhanced proliferation compared with 0.5 hour/day and control (fig. S11). These results suggest that 1 Hz for 1 hour/day provides effective cyclic mechanical cues that promote mechanotransduction without overloading the cells. Therefore, the optimal parameters for millirobot actuation were set to 1 Hz, 1 hour per day for all the following experiments.

Predominant green fluorescence and low red fluorescence demonstrate the dominant population of live cells in Fig. 2 (D to I). Figure 2J shows that cell viability is improved with mechanical stimulation, which indicates that millirobot actuation has minimal effect on cell survival. Meanwhile, myotube-like cell morphology (large and elongated cells) starts to appear in the millirobot actuation group on days 7 and 14, which indicates that mechanical stimulation may trigger the differentiation of C2C12 cells into myocytes. The influence of mechanical stimulation on cell proliferation in 2D cell-sheet biohybrid millirobot is investigated by cell counting kit-8 (CCK-8) assay on day 3, 7, and 14. Figure 2K and figs. S9 and S10 show that the millirobot actuation group has the highest absorption value compared to millirobot rest and control groups. The results indicate an up-regulation of cell proliferation for C2C12, hMSCs, and 3T3 cells when applying in situ mechanostimulation by millirobot actuation. Although the fluid flow induced by millirobot actuation contributes to nutrient and waste transport, the applied in situ mechanostimulation serves as the predominant factor responsible for the significantly enhanced cell proliferation observed in the 2D cell-sheet biohybrid millirobot (fig. S12). The millirobot actuation process of 2D cell-sheet biohybrid millirobot on day 0 and day 7 is shown in movie S2.

To optimize cell-sheet area and cell growth speed in a 2D soft perforated millirobot, we have investigated the influence of mechanical stimulation and different pore structures (hexagonal, square, and triangle) on cell growth. After seeding, cells adhere to the fibronectin cross-linked millirobot surface and start to proliferate until a dense monolayer has formed on all sides, including on the vertical pore walls. The formation of new tissue starts in the corners of the pores, while cells on the faces are initially resting. This growth behavior leads to a rounding of the corners and the formation of a round central opening (Fig. 2, L to Q), regardless of the original shape. The remaining open space reduces in diameter, gradually becoming filled by the cell sheet. Cell thickness in the corners is greater in the triangular pore followed by the square and then the hexagonal pore, that is, in the order of decreasing local curvature (fig. S13). The cell-sheet area on the pore, however, is greater for the hexagonal than the square one, followed by the triangular one on days 3 and 7 (Fig. 2R). Cell growth thickness (cell sheet area/pore perimeter) is increased over time and hexagonal has the highest cell growth thickness on day 7 (Fig. 2S). This cell growth behavior is in agreement with earlier tissue growth studies performed in macroscopic scaffolds with mouse preosteoblasts (*38*) and hMSCs (*39*) in static conditions.

The cell growth is due to the mechanical forces that arise in cells predominantly in regions of high curvature, which in turn stimulates the tissue growth (*40*). The main driving force behind this is the minimization of interfacial energy via the minimization of interfacial area. When applying in situ mechanostimulation induced by millirobot actuation as predicted in Fig. 1C, mechanical force and strain arise around the pore structure, leading to increased cell-sheet area and cell growth thickness of cell growth across all experimental groups (Fig. 2, O to Q). In the millirobot actuation group, the remaining pore spaces gradually decrease in diameter and become filled with tissue over the course of a 7-day culturing period, resulting in the formation of a 2D cell-sheet integrated biohybrid millirobot. In contrast, the pore spaces remain open in the millirobot rest group on day 7, as shown in Fig. 2 (N and Q). Similar cell-sheet growth is shown in 2D hMSCs-sheet biohybrid millirobots (fig. S14). Considering the highest cell-sheet area and growing thickness, hexagonal millirobot is chosen for the following studies.

### Effects of 2D biohybrid millirobot actuation on muscle cell functions

**Fig. 3.**
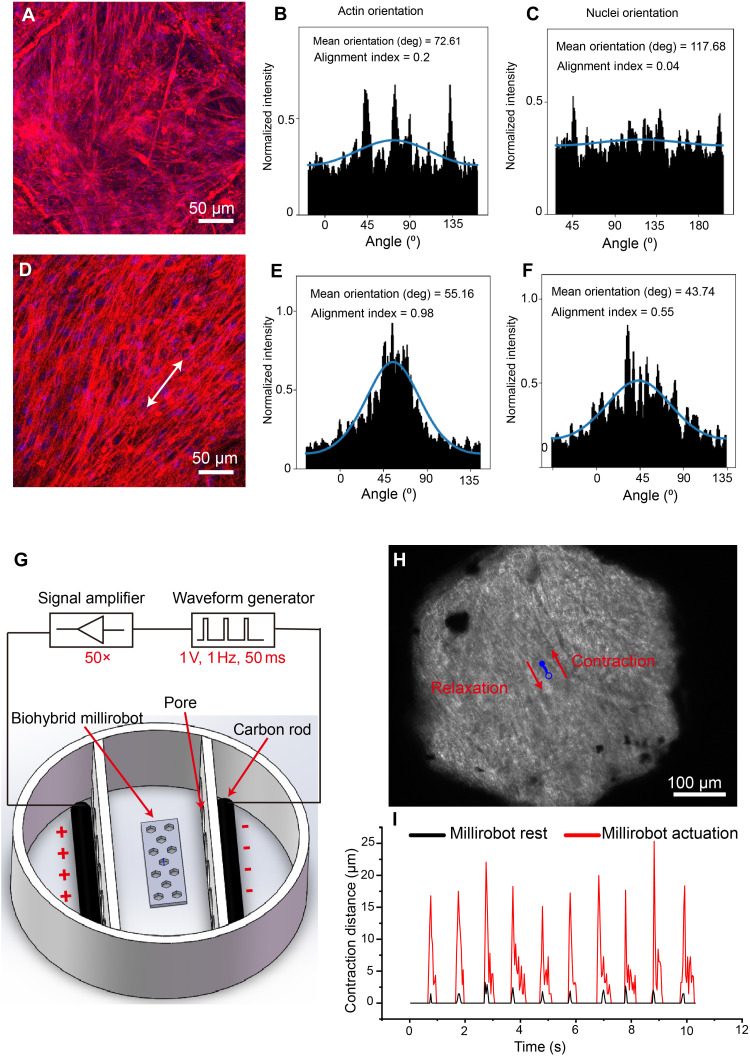
Effects of 2D cell-sheet biohybrid millirobot actuation on muscle cell orientation and contraction on day 28. (**A** to **C**) Millirobot rest group, (**D** to **F**) millirobot actuation group, and the arrow indicates the orientation of cell growth, (**G**) bipolar electrical pulse stimulation setup, (**H**) myotube contraction of 2D cell-sheet biohybrid millirobot in millirobot actuation group under electrical stimulation, and (**I**) contraction distance in millirobot rest and millirobot actuation groups under electrical stimulation.

Skeletal muscle is composed of highly ordered fibers of differentiated muscle cells, whose structure dictates functionality. In musculoskeletal myogenesis, aligning myoblasts in preparation for myotube formation is a crucial step (*41*). For cell-based therapy, efficiently organizing myoblasts/myocytes to form aligned myotubes in our biohybrid millirobots would positively affect muscle tissue repair. This may enhance the integration of delivered cells or tissues with the target tissue, leading to improved therapeutic outcomes. We have demonstrated that millirobot actuation promotes cell growth and survival. Next, we investigate whether in situ mechanostimulation induced by millirobot actuation influences skeletal muscle functions from morphology and molecular responses. The orientation of the actin stress fibers within cells neighboring the tissue border has been shown a parallel alignment with the tissue-fluid interface before the pores are filled with cells (*38*). After the pore structures are completely filled with cell sheet on day 28, actin fibers and nuclei exhibit a completely random orientation within the multicellular network in the millirobot rest group, as indicated by an alignment index of 0.2 and 0.04 (Fig. 3, A to C) (*42*). In contrast, in the millirobot actuation group, actin stress fibers and nuclei within the pore structures show a strong parallel alignment, with an index of 0.98 and 0.55 (Fig. 3, D to F).

It is known that mechanical interactions between cells and the environment modulate stress fiber abundance, structure, and organization (*43*). During millirobot actuation, the contraction and relaxation processes enable C2C12 cells to respond to in situ mechanostimulation, triggering the assembly of actin stress fibers to maintain focal adhesions in the direction of mechanical stress, resulting in a strong alignment of fibers and nuclei. Muscles being an electrogenic tissue, their development is closely linked to their electrical activity. Therefore, we are interested in looking at the contraction of these cells using an electrical pulse stimulator. First, we have developed a custom-designed setup capable of reliably stimulating excitable cells using a bipolar electrical pulse train (Fig. 3G). We leverage this stimulation method to synchronize the contraction of myotubes within the integrated 2D cell-sheet biohybrid millirobots. Electrical stimulation induces globally coordinated, strong twitch deformation of myotubes within the pore structure in the millirobot actuation group, whereas the millirobot rest group displays only weak contraction (Fig. 3H and movie S3). As shown in Fig. 3I and movie S4, the contraction displacement is 18.83 ± 2.80 μm in the millirobot actuation group, which is significantly higher than 2.01 ± 0.49 μm in the millirobot rest group, indicating that improved global actin stress fiber alignment has an observable positive impact on myotube function.

**Fig. 4.**
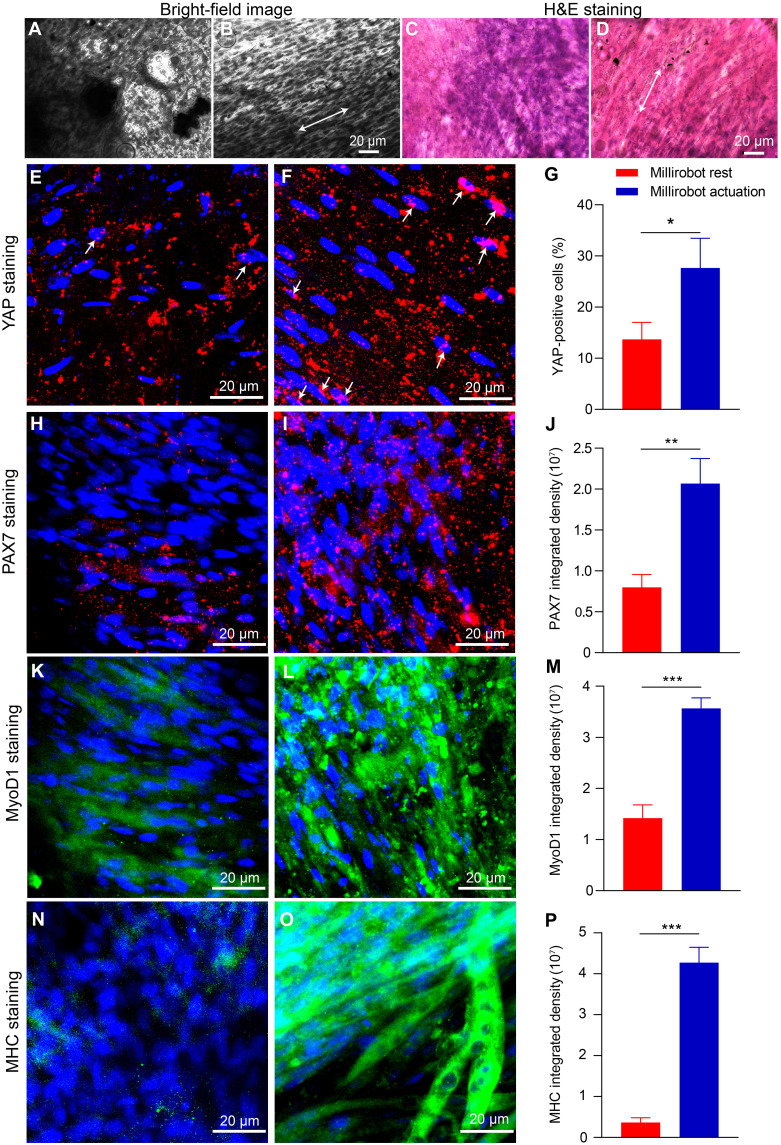
Histological and immunohistochemical analysis of the 2D cell-sheet biohybrid millirobot on day 28. (**A** and **B**) Bright-field images and the arrow indicates the orientation of cell growth; (**C** and **D**) H&E straining and the arrow indicates the orientation of cell growth; (**E** and **F**) YAP staining and the arrow indicates the YAP-positive cells showing nuclear colocalization; (**G**) fraction of cells positive for nuclear YAP; (**H** and **I**, **K** and **L**, and **N** and **O**) 3D projected images of PAX7, MyoD1, MHC protein staining; (**J**, **M**, and **P**) quantitative evaluation of PAX7, MyoD1, MHC immunofluorescence integrated density. [(A), (C), (E), (H), (K), and (N)] Millirobot rest while [(B), (D), (F), (I), (L), and (O)] millirobot actuation, *P < 0.05, **P < 0.01, ***P < 0.001.

Next, to better understand why millirobot actuation improves tissue contraction under electric stimulation, we investigate whether the in situ mechanostimulation generated by millirobot actuation could influence muscle cell differentiation and protein synthesis. In the group with millirobot actuation, cell alignment is visualized under bright-field microscopy and hematoxylin and eosin (H&E) staining, as shown in Fig. 4 (A to D). First, the potential of mechanical stimulation to enhance mechanotransduction pathways in skeletal muscle is assessed by examining the expression and localization of Yes-associated protein (YAP) (*44*, *45*). The 2D cell-sheet biohybrid millirobot, treated with in situ mechanostimulation over a 4-week period while maintaining cell immobilization demonstrates higher expression levels and nuclear localization of YAP compared to untreated cells (Fig. 4, E and F). The percentage of YAP-positive cells is significantly higher in millirobot actuation group than millirobot rest group (Fig. 4G). Chang *et al.* have shown that YAP functions as an upstream activator of c-Abl and Src tyrosine kinase, thereby activating the mitogen-activated protein kinase kinase-5 (MEK5)/extracellular signal–regulated kinase-5 (ERK5) pathway to promote C2C12 myocytes differentiation and myotube formation (*46*). Quiescent muscle stem cells express the paired-box transcriptional factor paired box 7 (Pax7) and, when activated, coexpress Pax7 with myogenic regulatory factors, such as myoblast determination protein 1 (MyoD1). Peter *et al.* demonstrated that Pax7 drives transcription in both quiescent and activated muscle stem cells and continues to do so in cells that subsequently cease myoblast to myocyte differentiation and myotube formation (*47*). Figure 4 (H to J) illustrates that the millirobot actuation group exhibited significantly higher PAX7 protein expression compared to the millirobot rest group on day 28. This suggests that millirobot actuation may positively influence cell proliferation during myoblast-myocyte differentiation.

The differentiation of myoblasts into terminally differentiated myocytes is tightly regulated by the coordinated actions of MyoD, Myf5, and myogenin, all of which belong to the basic helix-loop-helix transcription factor family (*48*). Upon the initiation of differentiation, MyoD interacts with myocyte enhancer factor 2 transcription factors to activate muscle-specific genes. This initial gene expression change is followed by cell cycle withdrawal, silencing of genes required for cell proliferation, and ultimately the formation of terminally differentiated myocytes (*49*). Figure 4 (K to M) demonstrates a significantly higher expression of MyoD1 protein in the 2D cell-sheet millirobot treated with millirobot actuation on day 28, compared to untreated cells. Figure 4 (N to P) reveals a notably increased level of myosin heavy chain (MHC) expression in the millirobot actuation group versus millirobot rest cells. These findings suggest that in situ mechanostimulation induces myocyte differentiation, which may explain the highly aligned myotube formation and improved electric stimulation performance observed in the 2D cell-sheet millirobot on day 28.

### Effects of 3D biohybrid millirobot actuation on muscle tissue formation

Skeletal muscles comprise bundles of densely packed, highly oriented muscle fibers embedded within a complex 3D extracellular matrix (*50*). This matrix provides essential structural support, guiding the organization and alignment of the muscle fibers while maintaining the integrity and overall architecture of the muscle tissue. For cell-based therapy to treat tissue defects, 2D cell-sheet biohybrid millirobots may have limitations in delivering large quantities of cells and tissues. To extend this approach to a 3D context, we have developed a 3D cell-laden hydrogel millirobot for the therapy of tissue defect repair. The influence of in situ mechanostimulation on cell and tissue functions within the 3D environment has also been investigated. We fabricate a sandwich-like 3D millirobot with a perforated sheet-shaped millirobot embedded within a cell-laden Matrigel composite hydrogel. The reversible contraction-relaxation transformation of the 3D cell-laden hydrogel millirobot, designed to mimic the muscle training process in 3D, can be repeatedly triggered by cyclic magnetic actuation, as demonstrated in movie S5.

**Fig. 5.**
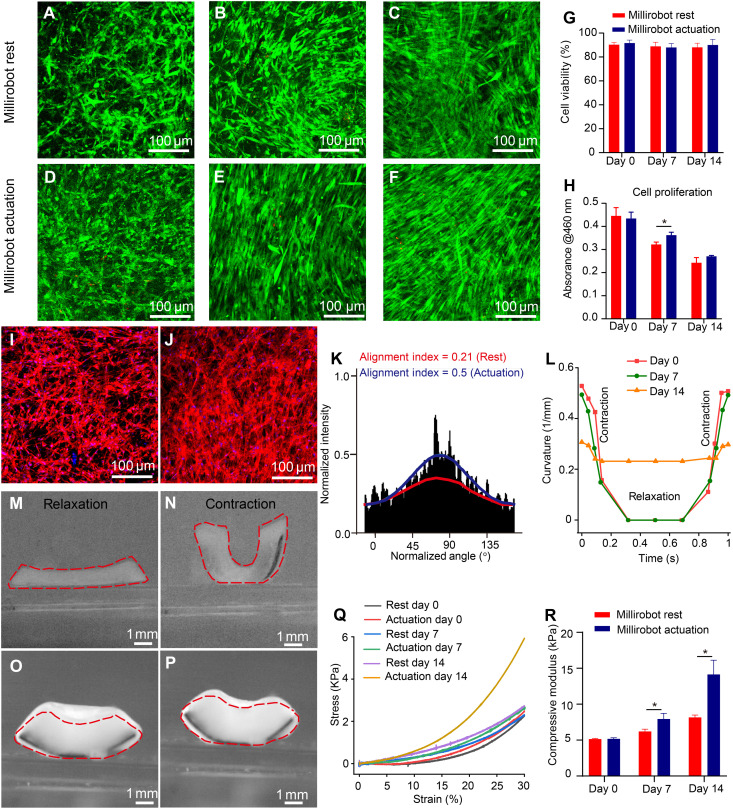
Effects of 3D cell-laden hydrogel millirobot actuation on muscle tissue formation. (**A** to **F**) Live-dead cells images in 3D cell-laden hydrogel millirobots on day 0 (A and D), day 7 (B and E), and day 14 (C and F) with millirobot rest (A to C) and actuation (D to F); quantitative analysis of cell viability (**G**) and cell proliferation (**H**); (**I** and **J**) actin staining on day 14 with millirobot rest (I) and actuation (J); (**K**) actin fiber orientation; (**L**) curvature change of 3D cell-laden hydrogel millirobots overtime under millirobot actuation; (**M** to **P**) bright-field images of 3D cell-laden hydrogel millirobots at the highest relaxation (M and O) and contraction (N and P) moments under magnetic actuation on day 0 (M and N) and day 14 (O and P); (**Q**) stress-strain curves; and (**R**) compressive modulus analysis of 3D cell-laden hydrogel millirobots under millirobot rest and actuation. *P < 0.05.

Predominant green fluorescence and low red fluorescence demonstrate the dominant population of live cells in Fig. 5 (A to F). Figure 5G shows that cell viability was not significantly different between millirobot rest and millirobot actuation groups (>85%). These results indicate that our cyclic millirobot training has minimal effect on cell survival, which is similar to our previous studies (*27*). On day 7, the CCK-8 activity in the actuation group is significantly higher than that in the rest group, which indicates that mechanical stimulation increases cell proliferation. Cell survival may benefit from the millirobot actuation on media transport (fig. S9) in the early culture period. An approximately 40% decrease in the average CCK-8 activity from day 0 to day 14 within the same groups is found after culture in C2C12 differentiation media for 14 days. The overall decrease is similar to that in our previous results (*51*), which may have resulted from the myogenic differentiation of C2C12 cells. Aligned cell morphology is observed in the millirobot actuation group on day 7 (Fig. 5, B and E) and day 14 (Fig. 5, C and F and I and J), in contrast to the random morphology seen in the millirobot rest group. The alignment index of actin fibers on day 14 is 0.5 in the training group, which is higher than the 0.21 observed in the no training group (Fig. 5K). This result is consistent with the alignment observed in the 2D cell-sheet biohybrid millirobot following the training process. However, the alignment index in the 3D cell-laden hydrogel is lower than in the 2D cell-sheet millirobot after millirobot actuation process, likely due to the omnidirectional conditions in the 3D cell culture environment.

Skeletal muscle hypertrophy typically occurs with repeated bouts of resistance exercise, and the resulting increase in muscle size is believed to be crucial for enhancing muscle function (*52*). Under magnetic actuation applying in situ mechanostimulation, the 3D cell-laden hydrogel millirobot demonstrates significant deformation on day 0 (Fig. 5, M and N), and the curvature change is larger than 0.5 in relaxation to contraction process. However, over the 14-day period of millirobot actuation, the curvature at contraction decreases while the curvature at relaxation increases (Fig. 5L). The curvature change during the contraction-relaxation cycle is about 0.07 on day 14, indicating a reduced deformation of the 3D cell-laden hydrogel millirobot under the same magnetic actuation from days 0 to 14 (Fig. 5, O and P). The decline in curvature changes over time may be attributed to two factors: the increase in hydrogel thickness and mechanical properties. The hydrogel thickness increased from 1.16 ± 0.13 mm on day 0 to 2.26 ± 0.17 mm on day 14 (Fig. 5, M to P, and fig. S15). Our results are consistent with previous observations of human skeletal muscle cells in a Matrigel/collagen scaffold, which showed a 12% increase in fiber diameter and a 40% increase in myofiber area under mechanical stimulation (*53*).

**Fig. 6.**
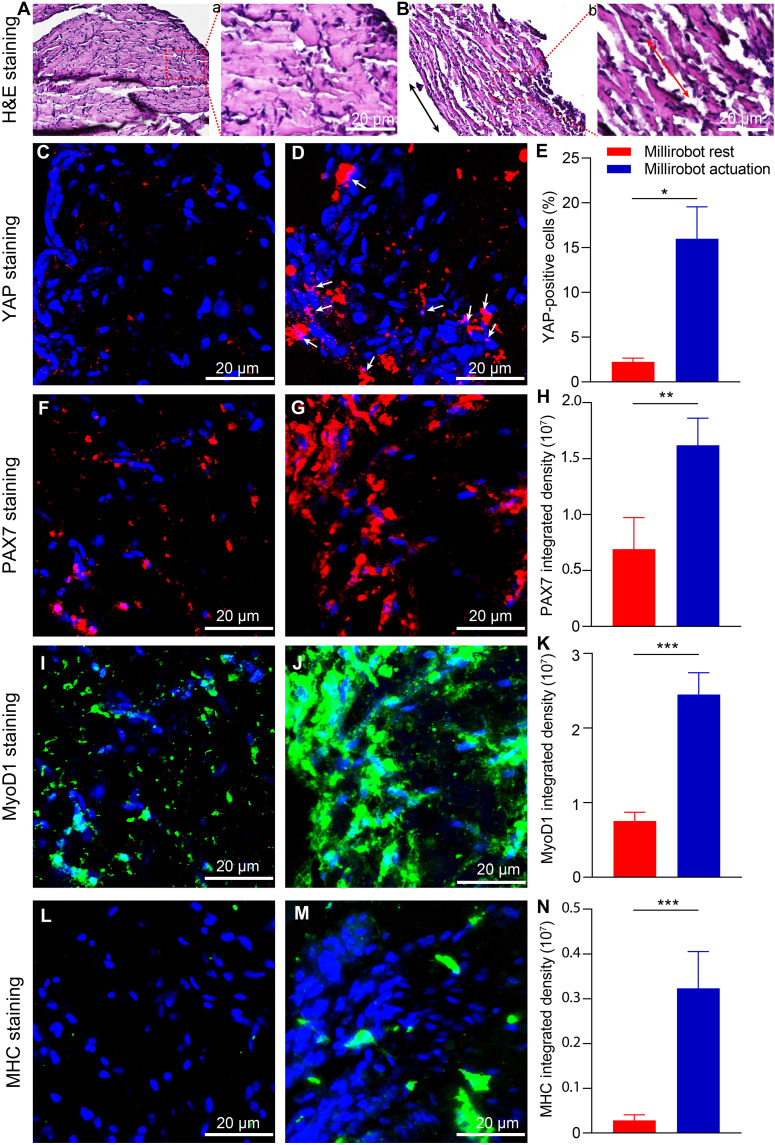
Histological and immunohistochemical analysis of the 3D cell-laden hydrogel millirobots on day 14. (**A** and **B**) H&E straining and (a and b) zoom-in images of the H&E staining and (**C** and **D**) YAP staining and the arrow indicates the YAP-positive cells showing nuclear colocalization. (**E**) Fraction of cells positive for nuclear YAP; (**F** and **G**, **I** and **J**, and **L** and **M**) 3D-projected images of PAX7, MyoD1, and MHC protein staining; (**H**, **K**, and **N**) quantitative evaluation of PAX7, MyoD1, and MHC immunofluorescence integrated density. [(A), (a), (C), (F), (I), and (L)] millirobot rest while [(B), (b), (D), (G), (J), and (M)] millirobot actuation, *P < 0.05, **P < 0.01, ***P < 0.001.

In addition, our findings indicate that the mechanical properties of the 3D cell-laden hydrogels improve over time because of the formation of the engineered skeletal muscle constructs. The compressive modulus was significantly higher in the millirobot actuation group compared to the millirobot rest group on days 7 and 14 (Fig. 5, Q and R). These findings suggest that millirobot actuation may enhance muscle tissue formation. To further investigate the effects of in situ mechanostimulation on the organization and maturation of engineered muscle tissues in vitro, histological and immunohistochemical staining are performed on sections from the millirobots on day 14. H&E staining reveals well-aligned myofibers in the group with millirobot actuation, while the millirobot rest group exhibits randomly oriented fibers on day 14 (Fig. 6, A, a, B, and b). In situ mechanostimulation leads to an up-regulation of YAP protein expression, with more pronounced expression and nuclear localization in the millirobot actuation group compared to the millirobot rest group (Fig. 6, C to E). Consistent with findings under 2D conditions, a significantly higher expression level of PAX7, MyoD1, and MHC proteins is observed in the millirobot actuation group compared to the millirobot rest group on day 14 (Fig. 6, F to N). These histological and immunostaining results indicate that in situ mechanostimulation induced by millirobot actuation enhances myoblast-to-myocyte differentiation and myofiber formation with high alignment in 3D. While mechanical stimulation appears to regulate and increase YAP expression, the detailed mechanisms by why YAP expression influences 3D engineered muscle tissue formation remain unclear.

### Image-guided biohybrid millirobot locomotion for cell delivery

**Fig. 7.**
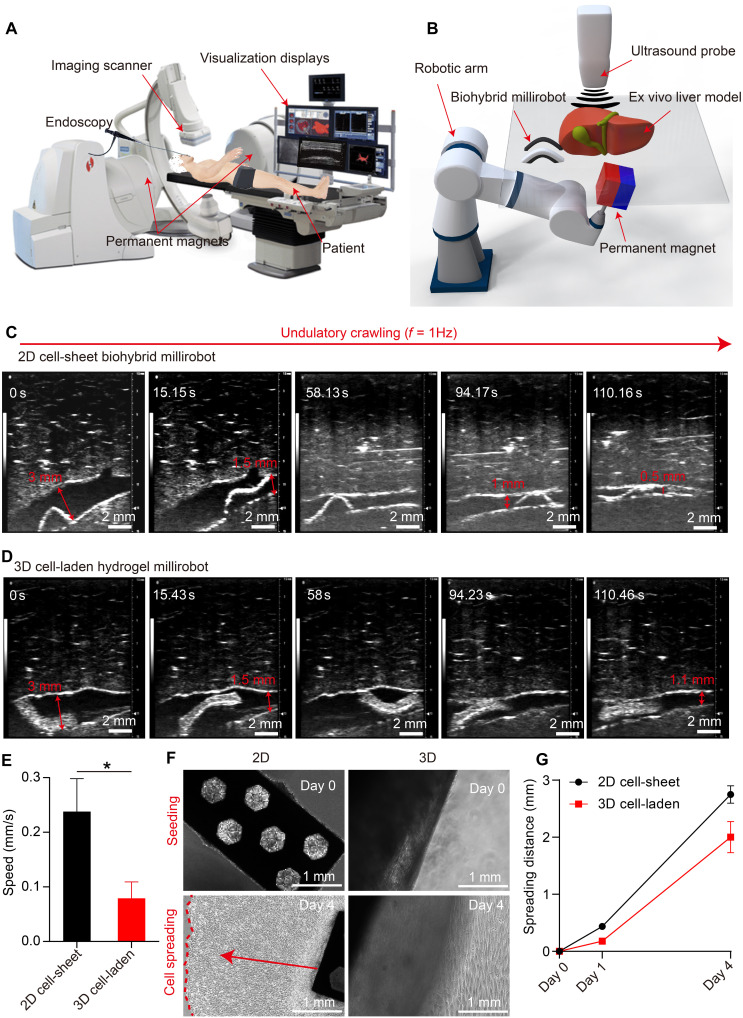
Image-guided biohybrid millirobot locomotion for cell delivery and spreading. (**A**) A clinical scenario of endoscopy-assisted magnetic actuation with a dual imaging system application for cell-delivery therapy, (**B**) an integrated robotic platform with magnetic actuation and US imaging system for millirobot delivery, (**C**) undulatory crawling of 2D cell-sheet biohybrid millirobot under magnetic actuation for target delivery from 3 mm to 0.5 mm of bile duct in an ex vivo pig liver model, and the arrow indicates the size of the bile duct, (**D**) undulatory crawling of 3D cell-laden hydrogel millirobot under magnetic actuation for target delivery from 3 mm to 1.1 mm of bile duct in an ex vivo pig liver model, and the arrow indicates the size of the bile duct, (**E**) locomotion speed for the 2D cell-sheet and 3D cell-laden biohybrid millirobots under the same magnetic actuation, *P < 0.05. (**F**) Cell migration from the biohybrid millirobots from day 0 to day 4 in vitro, and (**G**) cell spreading distance after delivery.

Low-strength and low-frequency magnetic fields–actuated millirobots are considered harmless to biological cells and can penetrate deep tissues, making this a promising approach for future clinical applications. Ultimately, the development of 2D cell-sheet and 3D cell-laden biodegradable millirobots provides a practical method for delivering cells and tissues to lesions within the human body. The millirobots can access both superficial tissues and deep-seated organs by traversing large cavities and narrow ducts. Figure 7A illustrates a conceptual drawing of such a system, termed endoscopy-assisted magnetic actuation with a dual imaging system. This system comprises a permanent magnetic actuation platform, an endoscope unit equipped with a catheter, and US medical imaging device. The integration of an endoscope with a millirobot allows for the rapid deployment and high-precision delivery of millirobots in real time to areas near lesions to avoid multiple organ barriers. The locomotion capability of the millirobot improves accessibility to hard-to-reach regions within internal organs that are typically beyond the reach of conventional endoscopic methods. To detect the real-time positions and deformations of the millirobots inside the ex vivo model, we have developed an ex vivo image–guided delivery system (*54*) integrating a controlled robotic arm with a permanent magnet that is compatible to magnetic actuation, alongside a US tracking system (Fig. 7B).

The development of 2D cell-sheet and 3D cell-laden biohybrid millirobots provides an optimal approach for delivering cells and tissues to lesions within the human body, whether at the superficial tissue level or deep within organs, including large cavities or narrow ducts. The soft and biocompatible biohybrid millirobots show adaptability to the surroundings by adaptive deformation. The cells can be harvested from the host so that the immune response may be minimized and even eliminated during millirobot delivery and therapy. In this study, we use the bile duct in an ex vivo pig liver model to demonstrate the cell delivery capability of 2D cell-sheet and 3D cell-laden biohybrid millirobots filled with phosphate-buffered saline (PBS) solution under magnetic actuation. The 3D cell-laden biohybrid millirobots have the potential application for inner tissue defect repair in cell-based therapy. The dimensions of the ex vivo pig bile duct model range from 3 to 0.5 mm. The sample size of the 2D cell-sheet biohybrid millirobot was 6.4 mm by 2 mm by 0.2 mm, while the 3D cell-laden biohybrid millirobot measured 7.5 mm by 4.2 mm by 1.1 mm for the millirobot locomotion experiment in an ex vivo pig liver model. The magnetic actuation system has been developed in previous study from our group (*54*). By rotating a cubic permanent magnet (50 mm) to provide a magnetic field in the *x*-*z* plane, the biohybrid millirobot achieves different deformation shapes to adapt ducts with different dimensions. Ren *et al.* demonstrated that a sheet-shaped robot can deform into a “C” shape when the robot is in a big gap, where the ratio between the robot length and the gap width (δ) is smaller than 2.6. When the robot comes into a small gap (δ > 2.6), a sinusoidal shape appears because of the squeezing of the upper and lower boundaries (*30*). With a rotating magnetic field, the robot exhibits locomotion capabilities in confined spaces with filling liquid (PBS). As shown in Fig. 7C and movie S6, the 2D cell-sheet biohybrid millirobot exhibits an undulatory crawling motion in the direction of the traveling body wave under a rotating magnetic field with a frequency of 1 Hz because of the friction between the millirobot and duct (*30*). The deformation shape changes from an inverted C shape to a sinusoidal shape as the duct dimension decreases from 3 to 0.5 mm. These results indicate that the 2D cell-sheet millirobot is capable of delivering cells to regions as narrow as 0.5 mm, which are beyond the reach of conventional endoscopy. Because of the larger thickness of the 3D cell-laden hydrogel millirobot (1 mm), it performs a sinusoidal shape within ducts ranging from 3 to 1.5 mm in diameter and encounters difficulty moving forward when the diameter is 1.1 mm (Fig. 7D and movie S7). The locomotion speed of the 2D cell-sheet biohybrid millirobot is 0.24 ± 0.05 mm/s (Fig. 7E), which is significantly higher than the 3D cell-laden biohybrid millirobot (0.08 ± 0.02 mm/s) under the same magnetic actuation. After completing the cell delivery process, the millirobots could also retract from the lesion site via magnetic actuation controlled by a robotic arm (movies S8 and S9).

To further verify the adaptability of the magnetic soft millirobots in complex curved ducts, we conducted ex vivo and in vitro locomotion experiments. In the ex vivo porcine liver model, the millirobots selectively navigated along different intrahepatic channels under a rotating magnetic field by adjusting the field orientation and position (movie S10). In the in vitro–curved PDMS duct model, the millirobots successfully passed through narrow and tortuous pathways with stable propulsion and controllable trajectories (movie S11 and fig. S16). These results demonstrate that the millirobots can efficiently adapt to multibranch and curved ducts, confirming their potential for targeted cell delivery in complex biological environments.

### In vitro controlled locomotion and cell spreading

Following the delivery of 2D cell-sheet and 3D cell-laden biohybrid millirobots to the lesion site in an ex vivo liver model, we hypothesize that the transplanted cells can migrate out of the millirobots to support local tissue regeneration. However, cell proliferation in the ex vivo liver is limited because of insufficient nutrient and oxygen supply. In our preliminary trials, we attempted a single-specimen ex vivo assay by delivering cells onto freshly excised mouse muscle and maintaining the construct under multiday incubation. Owing to the lack of perfusion in thick muscle tissue, ischemic degradation and subsequent contamination occurred within 48 hours, which prevented reliable proliferation assessment. Therefore, we decoupled the ex vivo delivery process from the in vitro proliferation and functional assays to evaluate cellular behaviors without the confounding effects of tissue necrosis or microbial contamination inherent to nonperfused organ culture.

To better visualize the cell migration process under favorable culture conditions, both 2D cell-sheet and 3D cell-laden biohybrid millirobots were deployed onto the surface of a petri dish. Under a magnetic field generated by a 12-mm cubic permanent magnet, the millirobots exhibited controlled locomotion, such as walking and rolling-toward designated regions on the dish surface (fig. S17 and movies S12 and S13). During locomotion, the biohybrid millirobots demonstrated adaptive seeding, cell spreading, and proliferation on the well plate surface (Fig. 7F and figs. S18 and S19). The C2C12 and hMSC cells cultured on the millirobots showed progressive spreading and proliferation during incubation (Fig. 7F and figs. S18, A to C, and S19), with the spreading area of C2C12 cells increasing linearly over time (Fig. 7G). Postmigration cell viability remained high, exceeding 95% on day 4 (fig. S18, D to F). Visual inspection of figs. S18 and S19 further revealed anisotropic migration behavior, where cells preferentially migrated from both longitudinal ends of the millirobot toward the culture substrate. Although this directionality has not yet been quantitatively analyzed, the end-directed migration trend appears consistent with the millirobot’s longitudinal curvature and the cellular alignment induced by in situ mechanical stimulation (Figs. 3, D to F, and 5, D to F). To further emulate in vivo–like interactions, we investigated the spreading and proliferation of C2C12 cells on a preformed cell layer following millirobot deployment (fig. S18, G to I). The migrated cells maintained high viability (> 95%) and successfully adhered to and proliferated on the preexisting cellular substrate, confirming their ability to integrate with surrounding tissue-like environments. Together, these findings demonstrate that cells can effectively migrate and spread from the biohybrid millirobots onto neighboring surfaces without the need for additional postdelivery treatments.

A single-sequence assay in which the biohybrid millirobots first locomote to predefined targets and then apply cyclic deformation at the target under the same magnetic field was performed. Both 2D cell-sheet and 3D cell-laden hydrogel robots exhibited stable, field-controlled walking/rolling followed by on-site mechanostimulation on ex vivo muscle tissue model (movies S14 and S15). These data confirm that navigation and localized mechanostimulation can be executed seamlessly in one operation.

### Effects of millirobot actuation on ex vivo tissue deformation

**Fig. 8.**
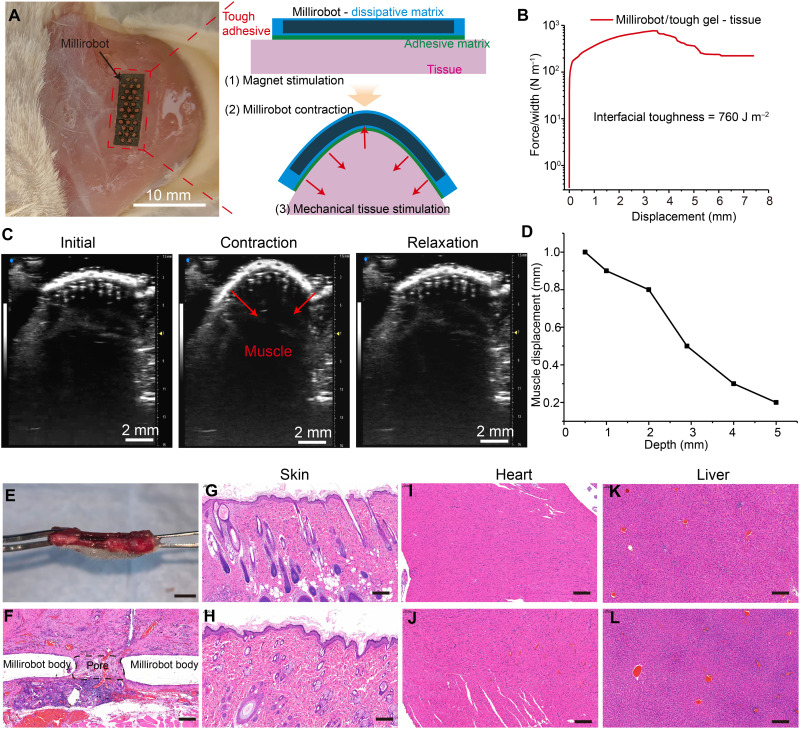
Ex vivo tissue deformation and in vivo toxicological assessment. (**A**) The adhesion of soft millirobot on the surface of mouse muscle tissue and a schematic illustration of a robust interface formation between elastomer, tough gel, and tissue; (**B**) curves of the peeling force per width versus displacement for the soft millirobot and tissue interfaces; (**C**) US images of ex vivo tissue deformation during actuation; and (**D**) estimation of muscle displacement as a function of tissue depth. (**E**) Image of millirobot implantation for 14 days; scale bar, 5 mm. (**F**) H&E image of millirobot-tissue integration in vertical direction. (**G** to **L**) H&E staining of major organs [the skin (G) and (H), heart (I) and (J), and liver (K) and (L)] after implantation for 14 days, (G, I, and K) millirobot implantation, (H, J, and L) control group; scale bar, 200 μm.

In addition to enabling in situ mechanical stimulation of cells in vitro, the perforated millirobots hold significant potential as active patches for mechanotherapy, offering targeted on-site mechanostimulation to cells and tissues. To demonstrate proof of concept, we design a 2D-perforated millirobot (13.2 mm by 3.4 mm by 1 mm) (fig. S20) that can be actuated by a magnetic field to perform reversible contraction-relaxation transformations on skeletal muscle tissue in an ex vivo mouse model. The millirobot-adhesive biohybrid is prepared by chemically anchoring the elastomer component of the millirobot to a hydrogel adhesive (*55*). The millirobot is embedded in a dissipative matrix and chemically bonded to the actuator’s elastomer using benzophenone. This dissipative matrix is anchored to the tissue with chitosan-bridging adhesive matrix (Fig. 8A). T-peel tests confirm a robust assembly of the millirobot-tissue interfaces, with adhesion strength measured at 760 J/mm^2^ (Fig. 8B), similar to previously reported results (*55*, *56*). To assess the ex vivo delivery of on-site mechanostimulation, the millirobot is adhered to the lateral gastrocnemius muscle of mice and actuated magnetically.

As shown in Fig. 8C, the millirobot is applied in situ mechanostimulation through cycles of contraction and relaxation of the muscle tissue under magnetic actuation. According to the Euler-Bernoulli beam theory, the compressive strain (ε) on the hydrogel surface is defined as

ε=−κy

The local curvature (κ) was quantified from the millirobot’s midline profile extracted from experimental images, while *y* denotes the distance from the neutral axis to the hydrogel surface. The corresponding compressive stress was calculated as

σ=Eε

where *E* represents the compressive modulus of the muscle tissue (*57*). The results indicate that, at maximum deformation, the millirobot applies a compressive stress up to 3.4 kPa on the muscle tissue. Real-time high-resolution US imaging confirms that the skeletal muscle beneath the millirobot deforms significantly along the actuation axis when the permanent magnet moved up and down (movie S16). Although the magnitude of tissue displacement decreases with increasing tissue depth, displacement is still observed at a depth of 5 mm within the muscle (Fig. 8D).

### In vivo toxicological assessment of millirobot implants

To evaluate the in vivo biocompatibility, degradation, and integration behavior of the soft millirobots, we performed subcutaneous implantation in rats and analyzed the implanted sites after 2 weeks by H&E staining (Fig. 8, E to L, and fig. S21). The acellular white region corresponded to the remaining millirobot body, while dense fibroblast proliferation and capillary formation were observed around and within the porous structure, without signs of inflammatory cell infiltration or tissue necrosis. The surrounding dermal tissue exhibited a continuous morphology and abundant microvessels, indicating a stable local blood supply. Host tissue effectively penetrated through the predesigned perforated channels, forming a continuous and integrated tissue-material interface (Fig. 8, E and F; movie S17; and fig. S22). The tissue-millirobot biohybrid exhibits similar magnetic actuation performance after in vivo implantation, indicating that demagnetization may not occur over time. Moreover, histological analysis of the overlying skin and major internal organs (heart, liver, spleen, lungs, and kidneys) revealed no pathological abnormalities compared with controls, with intact skin appendages and preserved organ morphology. These results demonstrate excellent local and systemic biocompatibility, showing that although PDMS and NdFeB@SiO_2_ are nondegradable, the porous and biofunctionalized architecture enables partial tissue ingrowth, vascularization, and long-term integration with surrounding tissues.

In hematological and biochemical analyses (fig. S21), routine blood parameters—red blood cells, white blood cells, hemoglobin, and platelets—remained within normal ranges and showed no statistically significant differences between the implant and control groups. In addition, serum biochemical markers for liver function (alanine aminotransferase and aspartate aminotransferase), kidney function (blood urea nitrogen), cardiac enzymes (cretine kinase), glucose, lipids, and total protein levels were all within physiological limits, indicating no signs of systemic toxicity. These findings confirm that the millirobot demonstrates excellent biocompatibility in vivo, with no detectable local or systemic toxicity, supporting its potential application in in vivo muscle tissue deformation.

## DISCUSSION

To enhance cell functionality and address the low targeting efficiency of current cell-based therapies, we have developed a magnetically driven, soft biohybrid perforated millirobot platform technology capable of providing in situ mechanostimulation to cells and facilitating local cell delivery. The in situ mechanostimulation for various cellular morphologies, including 2D, 3D, and tissue, is achieved through external magnetic actuation, mimicking muscle training exercises with cycles of contraction and relaxation. These soft perforated millirobots exhibit high biocompatibility, supporting cell growth within their pore structures, thus integrating C2C12 muscle cells, 3T3 fibroblasts, and hMSCs into the 2D cell-sheet biohybrid millirobots. The hexagonal structure offers the largest cell-sheet area and promotes cell growth thickness for 2D cell growth in vitro. As proof of concept, the 2D cell-sheet millirobot presents potential applications as active patches for mechanotherapy, delivering targeted on-site mechanical stimulation to cells and tissues in an ex vivo tissue model. For cell-based therapy aimed at treating tissue defects, 2D cell-sheet biohybrid millirobots may face limitations in delivering large quantities of cells and tissues. To extend this approach to a 3D context, we have developed a 3D cell-laden hydrogel millirobot for cell and tissue delivery. In addition, the biohybrid millirobot enables real-time transport and targeted delivery of cells and tissue to narrow regions within an ex vivo liver model through magnetic actuation and US imaging.

To investigate the effect of in situ mechanostimulation on cell functionality within 2D cell-sheet and 3D cell-laden biohybrid millirobots, we use C2C12 muscle cells as a model. The results demonstrate that mechanical stimulation significantly enhances the actin fiber orientation in both 2D and 3D cultures and improves the myotube contraction under electrical stimulation in the 2D cell-sheet biohybrid millirobot. This mechanical stimulation activates the mechanosensor YAP protein expression and its nuclear localization, enhancing myoblast-myocyte differentiation, as evidenced by the increased expression of PAX7, MyoD1, and MHC proteins. Furthermore, the perforated millirobot can apply on-site mechanostimulation under magnetic actuation to the muscle tissue surface. We develop an integrated robotic platform that combines magnetic actuation with US imaging to evaluate the precision of cell delivery capabilities for cell-based therapies. This platform enables rapid, high-precision delivery of biohybrid millirobots in real time to narrow regions (0.5 mm for the 2D millirobot and 1.1 mm for the 3D millirobot) in an ex vivo pig liver model. The 2D cell-sheet millirobot demonstrates a higher locomotion speed compared to the 3D cell-laden hydrogel millirobot. After delivery, the cells exhibit strong migratory and proliferative abilities. Overall, we have developed a magnetically driven soft biohybrid perforated millirobot with dual functions: in situ mechanostimulation to improve cell functionality and precise cell delivery.

Compared with existing microrobotic or mechanical-stimulation platforms, our magnetic soft millirobot represents a distinct advancement by synergistically integrating in situ mechanostimulation and localized cell delivery within a single, magnetically controllable system. Traditional microrobots primarily function as passive carriers for cell transport, often limited by low cell-loading capacity, insufficient environmental cues for maintaining cell functionality, and poor controllability in confined biological spaces. Likewise, conventional mechanical-stimulation systems, whether motor- (*28*), dielectric- (*58*), magnetic- (*59*), or pneumatic-driven (*60*), are generally bulky, static, and incompatible with in situ tissue environments. In contrast, our perforated, fibronectin-modified soft millirobot provides a highly cell-permissive structure and enables wireless, spatially confined actuation to deliver physiologically relevant cyclic strain to cells, enhancing their alignment, differentiation, and contractile performance. This biocompatible actuation mechanism avoids thermal and electrical damage and can be scaled from 2D cell sheets to 3D hydrogel constructs and ex vivo tissues. Furthermore, the integration of magnetic actuation with US imaging allows real-time, precision navigation and delivery of biohybrid millirobots into narrow or deep anatomical regions. Collectively, this dual-functional design offers a simple yet versatile strategy that bridges mechanobiology and targeted regenerative therapy, transforming cell-based treatments from passive transplantation into actively modulated, dynamic tissue rehabilitation, and establishes a previously unexplored direction for the development of intelligent biohybrid soft robots capable of both therapeutic delivery and functional regeneration.

However, several critical challenges must be overcome to advance biohybrid millirobots toward clinical application. (i) In this study, we demonstrated the proof of concept using a nondegradable material system, in which the soft robot remains functional after delivery. For clinical translation, however, the use of biodegradable and biocompatible materials is essential. Priority should be given to Food and Drug Administration–approved biodegradable polymers, such as poly(ε-caprolactone) and related copolymers, which can maintain structural integrity during the functional period and gradually degrade thereafter. The current millirobots are fabricated from PDMS and magnetic NdFeB particles, materials whose long-term biosafety has not yet been fully established. Although surface modification and encapsulation can improve short-term cytocompatibility, the intrinsic nondegradable nature of these materials may still lead to chronic inflammatory reactions at the implantation site. Soft biohybrid millirobots can also be retrieved using magnetic actuation, as demonstrated in movie S8 and S9. (ii) Complex robot actuation: Present soft millirobots primarily use a simple contraction-relaxation mode to apply in situ mechanostimulation to cells. While these millirobots have shown some capacity for complex deformations, the effects of various mechanical stimulations on cell functionality need further exploration. (iii) Developing an ideal “all-in-one” ex vivo platform that integrates targeted delivery, in situ mechanical stimulation, cellular proliferation, and self-adhesion within the same tissue sample remains highly challenging. Major limitations include physiological constraints, contamination risks, and mismatched cell-tissue compatibility. As a next step, we plan to address these challenges by using autologous (matched primary) cells or establishing in vivo models using immunodeficient hosts, which would allow long-term cell viability, proliferation, and tissue integration to be more accurately evaluated. Our biohybrid millirobots integrate dual functionalities: in situ mechanostimulation and cell delivery, broadening the potential applications of soft millirobots in both mechanobiology and cell-based therapies.

While our ex vivo liver and muscle models, together with preliminary in vivo biosafety analyses, establish feasibility for image-guided navigation and on-site mechanostimulation, controlled in vivo demonstrations are nontrivial. Outstanding needs include the following: (i) replacement of PDMS/NdFeB with biodegradable or magnetically retrievable constructs to mitigate long-term foreign-body risks; (ii) delivery of sufficient magnetic torque at depth under physiologic motion and flows; and (iii) multimodal imaging and closed-loop control compatible with actuation. Our ongoing work therefore integrates a biodegradable perforated architecture, autologous (or immunodeficient) cell sources, and endoscopy-assisted magnetic actuation with US tracking for pilot rodent and large-animal studies, including predefined retrieval/bioresorption end points. These steps will close the gap between our present ex vivo results and fully powered in vivo efficacy studies.

## MATERIALS AND METHODS

### Fabrication of 2D-perforated soft millirobots

NdFeB@SiO_2_ particles were mixed with uncured Sylgard 184 silicone (PDMS, Dow Corning) in a 1:10 base-to-curing agent ratio and a mass ratio of 1:1. The silica coating process is shown in the Supplementary Materials. The mixture was then poured onto a poly(methyl methacrylate) substrate with 250-μm-thick spacers, and a razor blade was used to scrape across the substrate to control the sheet thickness. The scraped mixture was cured on a hot plate at 90°C for 60 min. After curing, the sheet was cut into 6.4 mm–by–2 mm rectangular pieces with various patterned pore structures (triangular, square, and hexagonal) using a laser machine (LPKF ProtoLaser U3, LPKF Laser & Electronics AG), as shown in fig. S4. The sheets were carefully peeled off the substrate using tweezers and wrapped around a nonmagnetic rod with a diameter of 2 mm. During this process, water-soluble glue was applied to ensure the sheets formed a closed loop around the rod. The robots were then placed in a VSM and oriented such that the glued seams were aligned at 0° relative to the direction of the applied magnetic field. This orientation introduced a phase shift in the sinusoidal magnetization profile when the robots were subjected to a 1.8-T homogeneous magnetic field for 10 s under a VSM (MicroSense, E27). After magnetization, the robots were detached from the rods and thoroughly rinsed in an ultrasonic cleaner with deionized water (DI) until all glue residue was removed from the surface.

The magnetic torque $\tau_{m}$ (s) acting on each segment of the millirobot can be expressed as a function of its local magnetization *m*(*s*), its spatial orientation *R*(*s*), and the applied magnetic field *B*

$$\tau_{m}\left(s\right)=\left[0\;0\;1\right]\left[R\left(s\right)\;m\left(s\right)\times B\right]$$

where *s* denotes the curvilinear coordinate along the millirobot’s midline. The rotation matrix *R*(*s*) is determined by the local bending angle θ(s)

$$R=\left[\begin{array}{ccc} \text{cos}\theta & -\text{sin}\theta & 0\\ \text{sin}\theta & \text{cos}\theta & 0\\ 0 & 0 & 1 \end{array}\right]$$

The angle θ(s) was extracted from the experimentally observed midline profiles of the deformed robots. By integrating the torque density along one-half of the robot’s body—considering that torques on both sides are equal and opposite—we calculated the total actuation torque.

### Functionalization of 2D-perforated millirobots with fibronectin

To facilitate cell attachment, the surfaces of the soft perforated millirobots were modified by covalently binding fibronectin, following the methods described by Tan *et al.* (*61*) and Ehrig *et al.* (*35*). This process uses (3-aminopropyl) triethoxysilane (APTES) and glutaraldehyde (GA) as a cross-linker to immobilize fibronectin on PDMS. Before surface modification, the soft millirobots were sterilized by ultraviolet irradiation for 1 hour and then soaked in 70% ethanol for over 12 hours. They were subsequently dried at room temperature in a laminar flow cabinet. The surface treatment commenced with oxygen plasma activation using a Tergeo Plasma Cleaner (PIE Scientific LLC) for 180 s. Immediately following plasma treatment, the soft millirobots were immersed in a 3% APTES solution in DI water for 20 min and then thoroughly washed with DI water. Subsequently, the millirobots were incubated in a 1% GA solution in DI water for 40 min and washed again with DI water. Last, the millirobots were incubated with fibronectin in PBS at a concentration of 50 μg/ml for 1 hour in an incubator. After incubation, they were extensively washed with PBS to remove any unattached fibronectin residues.

### Finite element (FE) analysis

For the FE analysis, the deformation of the shapes under external magnetic fields was simulated using a user-defined element subroutine implemented in the commercial FE analysis software ABAQUS, based on an established model (*33*). The following input parameters were applied for all simulations: a shear modulus of μ = 45.36 kPa, a bulk modulus of K = 500 μ (chosen to approximate incompressibility due to the large bulk modulus), and a uniform external magnetic field (3 and 300 mT). The magnetization parameter used in the simulation was the experimentally measured value (M = 80 k A/m) for the perforated millirobot.

### Virtual particle tracing and calculation of the average velocity

The acquisition rate of the particle image velocimetry (PIV) images in this study is 500 Hz taken by the high-speed camera (PHANTOM), which can help us achieve time-resolved flow field measurements. We assume that the flow fields around the millirobot training under the magnetic actuation are 2D and can be reconstructed by tracing microparticles (diameter = 1 μm) seeded in water through cross-correlation. To obtain the flow velocity, with components *u* and *v* along the *x* and *y* directions, respectively, cubic interpolation is applied to interpolate the 2D velocity-gridded data collected from the PIV experiment at any location within the observation region. With the interpolated data of velocity fields, the vorticity is calculated by $w_{z}=\frac{\partial v}{x}-\frac{\partial u}{y}$ , and the average flow velocity *U* is calculated by $U=\sqrt[{2}]{u^{2}+v^{2}}$.

### Formation of 2D cell-sheet and 3D cell-laden biohybrid millirobots and magnetic actuation

The C2C12 cell line (ACC 565, mouse) and NIH-3T3 cell line (ACC 253, mouse) were obtained from DSMZ, while hMSCs derived from bone marrow (SCC034) were sourced from Sigma-Aldrich Life Science. All cell lines were cultured in T75 flasks with expansion media in an incubator (37°C, 5% CO_2_) according to the standard protocols provided by the manufacturers. Cells were harvested at 80% confluency, and those at passage 4 were used for subsequent experiments.

### 2D cell-sheet biohybrid millirobot

Cells were cocultured with 2D-perforated millirobots at a density of 0.5 million cells per millirobot. The millirobots were cultured in 24-well clear flat-bottom ultralow-attachment multiple-well plates (Costar). For the C2C12 cell culture, expansion media containing aminocaproic acid (1 mg/ml; Sigma-Aldrich) were added to the seeded cells on the millirobots 90 min after seeding. This was followed by a 3-day incubation period in a standard cell culture incubator (37°C, 5% CO_2_). Three days after seeding, the medium was switched to a differentiation medium consisting of Dulbecco’s modified Eagle’s medium (high glucose) supplemented with 10% (v/v) horse serum (Thermo Fisher Scientific), 1% (v/v) penicillin/streptomycin, 1% (v/v) ʟ-glutamine, aminocaproic acid (1 mg/ml), and insulin-like growth factor-1 (50 ng/ml; Sigma-Aldrich) to induce the differentiation of myoblasts into myotubes within the 2D cell-sheet millirobots. For the culture of 3T3 and hMSCs cells, both the expansion and differentiation media were prepared according to the protocols provided by the manufacturer. Initially, the cells were cultured in an expansion medium for the first 3 days and then switched to a differentiation medium starting on day 3. The media were changed every 2 days thereafter. Details on the fabrication of the magnetic actuation device are provided in the Supplementary Materials. Mechanical stimulation via magnetic actuation (1 Hz, 1 hour/day) of the millirobots was applied to the cells starting from day 1.

### 3D cell-laden hydrogel millirobot

A cell-laden ink solution was prepared by mixing C2C12 cells at a concentration of 10 million cells/ml with 60% (v/v) Matrigel (Corning), fibrinogen (4 mg/ml; Sigma-Aldrich), and thrombin (Sigma-Aldrich) at a concentration of 0.5 U/mg of fibrinogen (*62*, *63*). The 3D cell-laden hydrogel millirobots were fabricated using a sandwich-like approach. A PDMS boundary support (9.6 mm by 5.2 mm by 2 mm, inner diameter) was adhered to the surface of a 24-well clear flat-bottom ultralow-attachment multiple-well plate using a thermosensitive hydrogel of 10% gelatin solution. First, 30 μl of the cell-laden ink solution was pipetted to the bottom of the boundary support and incubated for 1 hour to allow for the gelation of the Matrigel. Subsequently, a 2D-perforated millirobot was placed on the surface of the hydrogel, and an additional 70 μl of the cell-laden ink solution was pipetted on top, followed by another 1 hour incubation to complete the gelation of the top Matrigel hydrogel. After gelation, expansion media containing aminocaproic acid (1 mg/ml) were added to the wells for cell culture. The PDMS support automatically detached from the hydrogel after 3 days of culturing in the incubator and was then removed from the media. Initially, the biohybrid millirobots were cultured in an expansion medium in the first 3 days for cell proliferation. Following this period, the medium was switched to a differentiation medium and we set this day as day 0. Mechanical stimulation was applied starting on the same day.

### Cell viability, growth, proliferation, and cell orientation analysis

Cell viability in the 2D cell-sheet and 3D cell-laden biohybrid millirobots with control, millirobot rest, and millirobot actuation were assessed using a LIVE/DEAD cell imaging kit (Thermo Fisher Scientific) on days 3, 7, and 14 for 2D millirobot and on days 0, 7, and 14 for 3D millirobot. Confocal microscopy (SP8, Leica) was used to visualize the living (green) and dead (red) cells. Cell viability was calculated at the percentage of living cells among total cells using the ImageJ (*27*, *51*). Cell-sheet growth bright-field images were visualized by the All-in-one Fluorescence Microscope (BZ-X800 Series, KEYENCE). Cell-sheet area with different pore structures and cell growth thickness were measured with the ImageJ (*38*). Cell proliferation was assessed using a CCK-8 (WST-8/CCK-8, Abcam) at each time point. Culture medium (360 μl) and 40 μl of CCK-8 solution were added to each well and incubated at 37°C for another 1 hour. An aliquot of 100 μl was taken from each well and transferred to a fresh 96-well plate. The light absorbance of these samples was measured at 460 nm with a microplate reader (Bio-Rad 680, USA).

### Actin staining

After 28 days for 2D cell-sheet biohybrid millirobots and 14 days for 3D cell-laden biohybrid millirobots cultured in differentiation media, millirobots with millirobot rest and millirobot actuation groups were fixed in 4% formaldehyde in PBS for 1 hour. Millirobots were permeabilized in 0.1% Triton X-100/PBS solution for 30 min. Whole constructs were stained with phalloidin (1:400) and Hoechst (1:500) for 60 min to visualize filamentous actin and cell nuclei, respectively. Cells were visualized on a confocal microscope (SP8, Leica). Actin fiber orientation was analyzed with FiberFit developed by Northwest Tissue Mechanics Laboratory (*42*).

### Electric pulse stimulation

The contraction of myotubes was achieved by stimulating the cells with a pulse train using a custom-built electrical setup. We generated an alternative-current square wave as electrical pulses using a function generator (Tektronix AFG1022) and amplifier (50×, Trek 2100HF Series) and applied the electrical pulses to carbon electrodes placed around the 2D cell-sheet biohybrid millirobot, as shown in Fig. 3G. To avoid the effects of bubbles generated during electrolysis, a custom-designed device was built to separate the bubbles. Millirobots were stimulated with bipolar electrical pulses of 1 V, 1 Hz, and 50-ms pulse width. Top-view movies were required with the All-in-one Fluorescence Microscope (BZ-X800 Series, KEYENCE) with movie record at 30 frames/s. Contraction distance was analyzed with the TrackMate function in ImageJ.

### Mechanical properties

The mechanical properties of 3D cell-laden hydrogel millirobots were characterized by the measurement of the compressive modulus. The compressive modulus was assessed on an Instron instrument (3342, load cell of 10 N) at room temperature. Unconfined uniaxial compression tests were performed under displacement control, with a strain rate of 1 min^−1^ until 30% maximal deformation of the construct was reached. The 3D cell-laden hydrogel millirobots (*n* = 3) were tested after being cultured with the cell culture media in the incubator for 0, 7, and 14 days. The compressive modulus was calculated from the linear region of the stress-strain curve, which was between 5 and 15% deformation for all samples.

### Histology and immunohistochemistry staining

After 28 days of millirobot actuation, 2D cell-sheet biohybrid millirobots were fixed in 4% formaldehyde solution overnight and then dehydrated with a graded ethanol solution. Similar procedures were performed with the 3D cell-laden hydrogel millirobot after 14 days of actuation and embedded in kerosene wax. The 7 μm of hydrogel section was used in further experiments. After rehydrating the sections with a series of 100, 90, 80, and 70% ethanol solutions and a water solution, H&E staining was performed to visualize the cell/tissue orientation. The 2D cell-sheet millirobots and sections were stained with hematoxylin for 5 min, washed with running water for 5 min, and exposed to eosin solution for 2 min. The samples were then dehydrated and cleared in three changes of xylene for 2 min per change. A drop of mounting media was placed over the sections on each slide and a coverslip placed on top.

For immunohistochemical staining, the samples were permeabilized and blocked with PBS containing 0.3% Triton X-100, 1% bovine serum albumin, and 5% goat serum for 1 hour. The following primary antibodies were used for immunohistochemistry: YAP antibody (1:100, ab205270, Abcam), anti-MHC/MHC antibody (A4.1025) (1:100, Sigma-Aldrich), anti-MyoD1 antibody (5.2F) (1:100, ab16148, Abcam), and anti-PAX7 antibody (1:100, ab187339, Abcam). Secondary antibodies used were goat anti-mouse immunoglobulin G (IgG) H&L (Alexa Fluor 488) (1:1000, ab150113, Abcam) and goat anti-rabbit IgG H&L (Alexa Fluor 647) (1:1000, ab150079, Abcam). Nuclei were stained with Hoechst (1:400, ab145597, Abcam). Protein expression images were visualized under the SP8 Leica confocal microscope. Image processing was performed and calculated by ImageJ.

### Cell delivery and cell spreading

The magnetic actuation system was developed in our previous study (*64*). The US system (Vevo 3100, FUJIFILM Visualsonics Inc.) was used to visualize and record the locomotion of 2D cell-sheet and 3D cell-laden biohybrid millirobots. The US probe was held by another linked robotic arm (Panda, Franka Emika GmbH) (*54*). The ex vivo pig fresh liver model was ordered from the local slaughterhouses and washed several times with water to remove the mucous. Cell migrating experiments were performed in an in vitro experiment. Millirobots were actuated with the rotating magnetic field and seeding on the well plate and cell film. The cell spreading distance was visualized by the All-in-One Fluorescence Microscope (BZ-X800 Series, KEYENCE) with the bright-field image. The locomotion speed and cell spreading distance were analyzed by ImageJ.

### Millirobot actuation in an ex vivo model

The 2D-perforated millirobot with 13.2 mm of length, 3.3 mm of width, and 0.8 mm of thickness was performed the robot training for the ex vivo muscle tissue in female C57BL6/J mice and rat (Anatomy and Cell Biology Laboratory, Ulm University, Germany). The millirobot was embedded in the tough hydrogel following an established method (*55*, *56*). More detailed information was shown in the Supplementary Materials. For adhesion to the surface of muscle tissue, we added an adhesive polymer (chitosan) between tough hydrogel and muscle tissue. Briefly, 4% chitosan (medium viscosity, Sigma-Aldrich) and coupling reagents [24 mg/ml of 1-ethyl-3-(3-dimethylaminopropyl) carbodiimide and *N-*hydroxysulfosuccinimide] were mixed at a 1:1 ratio by vertexing. The mixture was then quickly applied to the surface of the tough hydrogel and was gently compressed on the millirobot for 5 min to achieve tissue adhesion.

Adhesion performance was quantified as the adhesion energy, the amount of interfacial energy required to propagate a unit area of interfacial crack. The adhesion energy was measured by T-peeling tests. The free ends of the millirobot-hydrogel and tissue were fixed to the grips of the machine. Unidirectional tension was applied with an Instron instrument (3342, load cell of 10 N) while recording the force and displacement. The loading rate was kept constant at 1 mm/min. The adhesion energy was determined as the force peak divided by the width of the sample.

After bonding on the surface of the muscle tissue, the millirobot was covered with US gel (Aquasonic). The mouse’s body was placed on the surface of the substrate, and a permanent magnet was traveled up and down to apply magnetic stimulation for the millirobot under the substrate with 1-Hz frequency. The US probe was held by the robot arm on top of the US gel to visualize the muscle contraction induced by the millirobot contraction and relaxation. Muscle displacement was analyzed by ImageJ.

### In vivo implantation

Male Sprague-Dawley rats (150 ± 10 g) were obtained from Chengdu Dashuo Bio-Technology Co. Ltd. (China). All animal experiments were conducted in accordance with the guidelines of the Medical Ethics Committee of West China Stomatology Hospital of Sichuan University (approval no. WCHSIRB-D-2022-459). The animals were housed under specific pathogen–free conditions, fed a standard laboratory diet, maintained on a 12-hour/12-hour light/dark cycle, and allowed at least 1 week of acclimatization before the experiments.

Sprague-Dawley rats were randomly assigned to two groups, fasted overnight with free access to water, and anesthetized with 1% pentobarbital sodium. The experimental group received millirobot implantation into the subcutaneous tissue of the back, while the control group underwent no implantation for 14 days. At the end of the study, the animals were euthanized, and their complete blood count serum and biochemical parameters were measured. Major organs (skin, heart, liver, spleen, lung, and kidney) were harvested and subjected to H&E staining for histological analysis. To ensure blinding, different personnel performed the surgeries and subsequent analyses.

### Statistical analysis

GraphPad Prism 9 was used to do the statistical analysis for the obtained data. The comparison of data for millirobot rest and millirobot actuation groups (*n* ≥ 3) at one-time points was done using a two-way analysis of variance (ANOVA) test together with pair-wise comparison, followed by Tukey corrections. The comparison of data for millirobot rest and millirobot actuation groups at different time points was done using a two-way ANOVA test together with Tukey’s multiple comparisons test. *P < 0.05 were considered statistically significant.
