# Supplementary material for: In situ mechanostimulation of biohybrid millirobots for enhanced cell functionality and delivery
Source: Sci Adv. 2026 Jan 2;12(1):eadx9616. doi: 10.1126/sciadv.adx9616 (PMC12758528; doi:10.1126/sciadv.adx9616)
Supplement: Supplementary file 1 — Supplementary Text Figs. S1 to S22 Legends for movies S1 to S17 [file sciadv.adx9616_sm.pdf]

Science Advances
AAAS

Supplementary Materials for

In situ mechanostimulation of biohybrid millirobots for enhanced cell functionality and delivery

Jianhua Zhang *et al.*

Corresponding author: Jianhua Zhang, jianhua.zhang@zju.edu.cn; Ziyu Ren, renzy@buaa.edu.cn; Metin Sitti, sitti@is.mpg.de

Sci. Adv. **12**, eadx9616 (2026)
DOI: 10.1126/sciadv.adx9616

The PDF file includes:

Supplementary Text
Figs. S1 to S22
Legends for movies S1 to S17

Other Supplementary Material for this manuscript includes the following:

Movies S1 to S17

Experimental Section

Silica coating: NdFeB magnetic microparticles (MQP-15-7, Magnequench, average diameter, 5 μm) were encapsulated with a silica (SiO_2) layer using the Stöber method (*29*), involving hydrolysis and polycondensation of tetraethyl orthosilicate (TEOS, Sigma-Aldrich). Initially, 40 g of NdFeB microparticles were dispersed in 1000 ml of ethanol and stirred vigorously at 1500 rpm using a digital mixer (Cole-Parmer) to prevent sedimentation. Subsequently, 60 ml of 29% ammonium hydroxide was slowly added, followed by the gradual addition of 2 ml of TEOS. The reaction mixture was stirred for 12 hours at room temperature, and then washed multiple times with acetone. The resulting suspension was vacuum-filtered to obtain the silica-coated particles (NdFeB@SiO_2).

Magnetic actuation: The magnetic actuation device consists of a magnetic platform, a motor, and a crank arm linkage, as shown in Figure S4. The bipolar stepper motors (part number 1528-1062-ND), which are powered by a 12V DC supply through the circuit board, were ordered from Adafruit Industries LLC. The cycle frequency was set to 1 Hz by modifying the serial number in the Arduino software. The platform, base, and linkage were printed using a Formlabs printer, and all components were assembled by hand.

Millirobot-tough hydrogel: Brief, millirobot was thoroughly cleaned with methanol and deionized water, and dried, and then was treated by absorbing benzophenone (10% in ethanol, 5 min). The millirobot actuator was washed two to three times with methanol and completely dried. Tough hydrogels were prepared by mixing an aqueous pre-gel solution (12% acrylamide, 2% sodium alginate (medium viscosity, Sigma-Aldrich), 0.037% N, N'-methylenebisacrylamide, 0.2% Irgacure 2959 photoinitiator) with ionic crosslinker (35 mM calcium chloride). The mixture was then quickly poured into a PDMS mold with a spacing of 1 mm, and pressed the millirobot embedded into the tough hydrogel. The hydrogel was assembled with benzophenone-treated millirobot followed by UV irradiation for 30 minutes to crosslink the polyacrylamide

network and to form bonds with the millirobot. The hydrogel-millirobot were then gently removed from the mold.

Results and discussion:

Structural stability and anticorrosion ability of magnetic NdFeB@SiO_2 particles

To assess the structural stability of NdFeB@SiO_2 particles, we examined their surface morphology using scanning electron microscopy (SEM) and performed elemental mapping via energy-dispersive spectroscopy (EDS) before and after 21 days of *in vitro* culture (Figure S2). The silica shell was found to be homogeneously distributed on the particle surfaces, and no noticeable changes in morphology were observed after culture. The total weight fraction of Si and O remained comparable before (5.3 ± 0.3%) and after (4.7 ± 0.4%) 21 days, indicating good structural stability during *in vitro* culture.

The anticorrosion performance of the silica coating was evaluated through a leaching test using a weak acidic solution (0.2 mM HCl, pH 3) on particles before and after 21 days of culture. No visible changes were observed at day 3, demonstrating the long-term protective effect of the silica shell under cell culture conditions (Figure S3). Furthermore, all cultured particles retained their magnetic response and were readily attracted to a magnet, showing no difference compared to particles prior to culture. These results indicate that NdFeB@SiO_2 particles maintain both structural integrity and magnetic properties without requiring remagnetization after 21 days of *in vitro* culture.

Effects of Millirobot Actuation on Fluid Transport in *In Vitro* Culture

The flowing interstitial fluid, which constitutes up to 20% of body mass, is distributed throughout the extracellular matrix, filling the spaces within tissues. This fluid provides cells with nutrients, facilitates waste removal, and plays a crucial role in microcirculation (*34*). To replicate a flowing-state fluid environment, the millirobot performs cyclic contraction-relaxation deformations that induce motion in the surrounding culture medium, thereby enhancing media mixing and facilitating localized fluid transport, which in turn benefits nutrient and waste exchange (Figure S7). To better visualize fluid transport and

calculate fluid velocity around the millirobots during magnetic actuation, we track virtually massless particles in flow fields using particle image velocimetry (PIV) experiments. Figure S8 shows the formation of two opposite vorticities on the two-sided surface of the millirobot at the moments of greatest contraction and relaxation. The 2D millirobot exhibits stronger vorticity compared to the 3D millirobot, attributed to a larger beating amplitude. Fluids are transported along both the x and y directions during millirobot actuation. The average velocity measured along cut line 2 over three actuation cycles show a pulsatile pattern with peak values of 1.25 mm/s (2D millirobot) and 1.01 mm/s (3D millirobot). The average velocity along cut line 1 is lower than that along cut line 2, indicating a stronger flow on the two sides compared to the middle part of the millirobot. The generation of velocity gradients and vortex flow waves along the millirobot enhances fluid transport and mixing.

Dye-tracing experiment for mimicking nutrient/waste transport visualization

To simulate the nutrient and waste exchange process driven by cyclic millirobot actuation, we introduced a droplet of colored dye near the millirobot and compared its distribution under actuated (0.6 Hz) and non-actuated conditions. As shown in the Figure S7, the actuated millirobot generated convective flows that actively mixed the surrounding dye, whereas in the control group, the dye spread only slowly through passive diffusion with minimal dispersion. This experiment demonstrates that the millirobot's actuation can effectively overcome diffusion limitations, thereby enhancing the delivery of fresh nutrients and the removal of metabolic waste. Although the test was conducted at 0.6 Hz, a higher actuation frequency (1 Hz) used in the cell experiments is expected to further improve diffusion efficiency.

Cell proliferation in the wellplate under induced fluid motion

Cells were cultured in a wellplate with a millirobot actuated in the same medium to generate fluid flow. No cells were cultured on the millirobot surface. Cell proliferation of C2C12, measured by CCK-8 assay (Figure

S12), showed no statistically significant increase compared to the static control group (millirobot rest). Although millirobot actuation successfully induced convective flow and enhanced local mixing in the dye diffusion test, this effect did not translate into a statistically significant increase in C2C12 cell proliferation in the wellplate. Several factors may account for this observation. First, the flow field generated by the actuated millirobot was relatively weak and localized, primarily enhancing mixing in the immediate vicinity rather than producing uniform medium circulation across the entire well. As a result, the overall nutrient and oxygen supply to the cells on the wellplate remained largely unaffected. Second, since the cells were not directly attached to the millirobot surface, the mechanical and biochemical stimuli induced by actuation were indirect and mild, insufficient to trigger a measurable proliferative response. Third, the low actuation frequency (1 Hz, 1 hour per day) and short stimulation duration likely limited the cumulative transport enhancement effect.

Image-guided Biohybrid Millirobot Locomotion for Cell Delivery

Ex vivo liver model experiment (Video S10): To evaluate the controllability and adaptability of the millirobots in a realistic biological environment, we performed an ex vivo experiment using a porcine liver duct model. By precisely adjusting the direction and position of the external rotating magnetic field, the millirobots were able to selectively navigate along different intrahepatic channels. The robots demonstrated smooth and reversible movement within the branching bile-like ducts, maintaining stable motion without blockage or structural damage to the tissue. This result confirms that the millirobots can adapt to multi-branch and variable-diameter channels in biological tissues.

In vitro curved duct experiment (Video S11): To further verify the ability of the millirobots to move through curved and narrow channels, we designed an *in vitro* PDMS model that mimics a narrow and tortuous microchannel environment. Under the actuation of a rotating magnetic field, the millirobots successfully

navigated through multiple curved pathways with different curvature radii from 3mm to 1.5mm, maintaining consistent propulsion and directionality. The motion trajectory was fully controllable by modulating the magnetic field vector, demonstrating the robots' adaptability to complex curved geometries. Together, these results provide direct experimental evidence that the magnetic soft millirobots are capable of stable locomotion in both biological and biomimetic curved duct systems, confirming their potential for targeted cell delivery in complex *in vivo* environments.

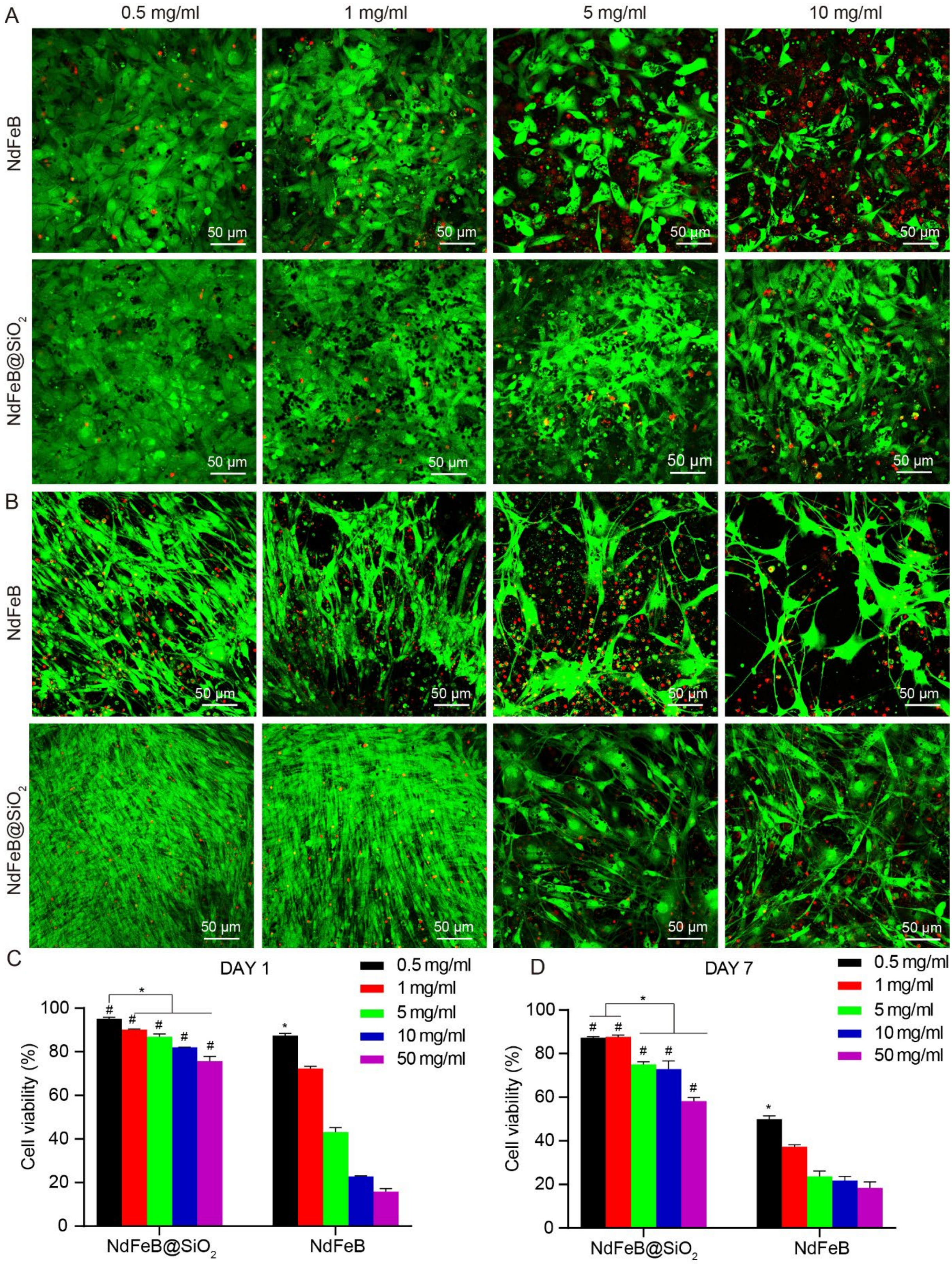

Figure S1. Biocompatibility of different concentrations of NdFeB and NdFeB@SiO2 magnetic particles in co-culture with 3T3 fibroblasts. (A, B) Live/dead staining images on day 1 and day 7, (C, D) quantitation of 3T3 fibroblasts viability after co-culturing with different concentrations of NdFeB and NdFeB@SiO_2

magnetic particles from 0.5 mg/ml to 50 mg/ml on day 1 and 7. # $P < 0.05$ indicates significant differences between the same concentration of NdFeB@SiO_2 and NdFeB magnetic particles on the same day. * $P < 0.05$ indicates significant differences between the 0.5 mg/ml group and the other groups on the same day. # $P < 0.05$ indicates significant differences between the NdFeB@SiO2 and NdFeB groups at the same concentration on the same day.

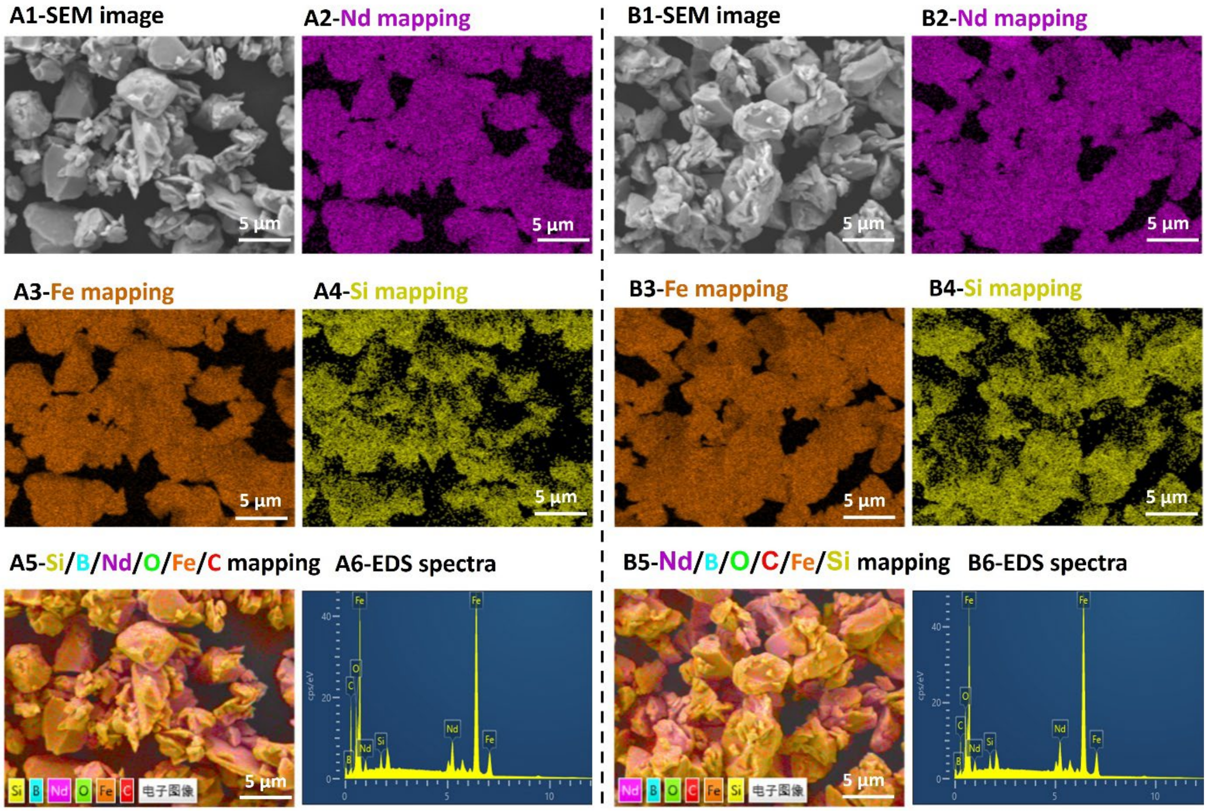

Figure S2. SEM images and EDS analyses of the surface morphology of NdFeB@SiO_2 particles before (A) and after 21 days (B) of *in vitro* culture. (A1, B1) SEM images; (A2, B2) Nd element mapping; (A3, B3) Fe element mapping; (A4, B4) Si element mapping; (A5, B5) combined Si/B/Nd/O/Fe/C element mapping; (A6, B6) EDS spectra.

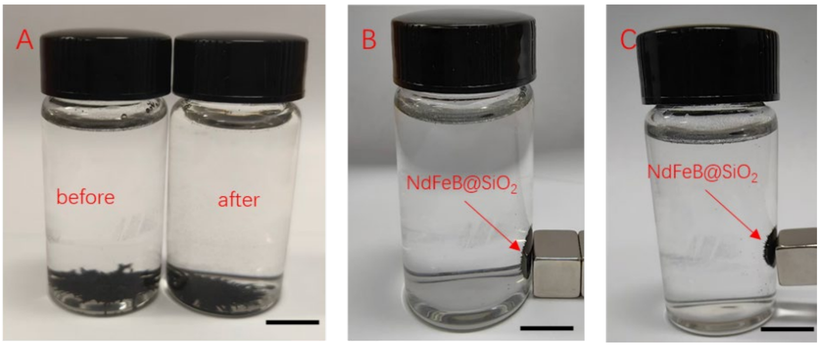

Figure S3. Leaching and magnetization tests. (A) Particles were cultured in a weak acidic solution (0.2 mM HCl, pH 3) before and after 21 days of cell culture. (B) Magnetization test of NdFeB@SiO_2 particles. (C) Magnetization test of NdFeB@SiO_2 particles after 21 days of cell culture. Scalar bar 10mm.

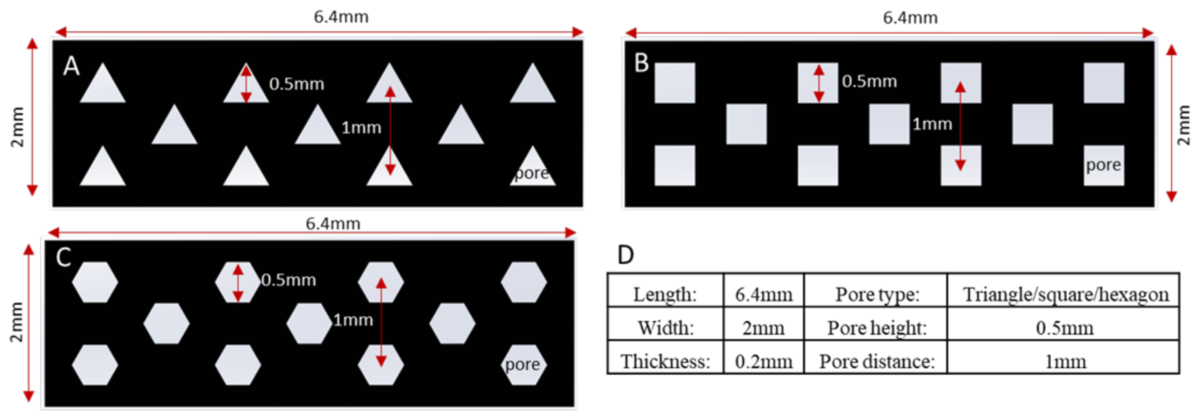

Length:	6.4mm	Pore type:	Triangle/square/hexagon
Width:	2mm	Pore height:	0.5mm
Thickness:	0.2mm	Pore distance:	1mm

Figure S4. 2D soft perforated millirobot design with different pore structures. (A) Triangle, (B) square, (C) hexagon, (D) sample size parameters.

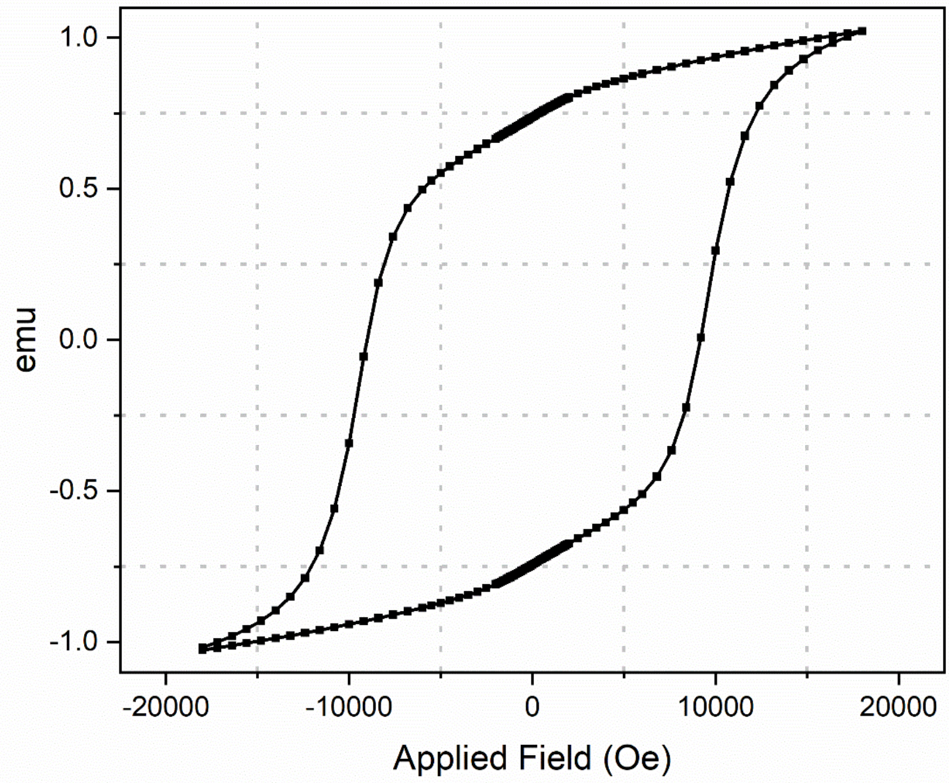

Figure S5. Magnetic hysteresis characteristics of millirobot materials, which is obtained by a vibrating sample magnetometer (VSM) under varying magnetic fields. The sample is made from PDMS (10:1), mixed with NdFeB@SiO_2, having a mass ratio of magnetic particles to PDMS at 1:1. The volume of this sample is 9.55 mm^3, the average remanent magnetization (Mr) at H=0 is 740.75 E^{-3} emu. Therefore, the magnetization magnitude is 80.95 ± 5.07 kA/m (n=4).

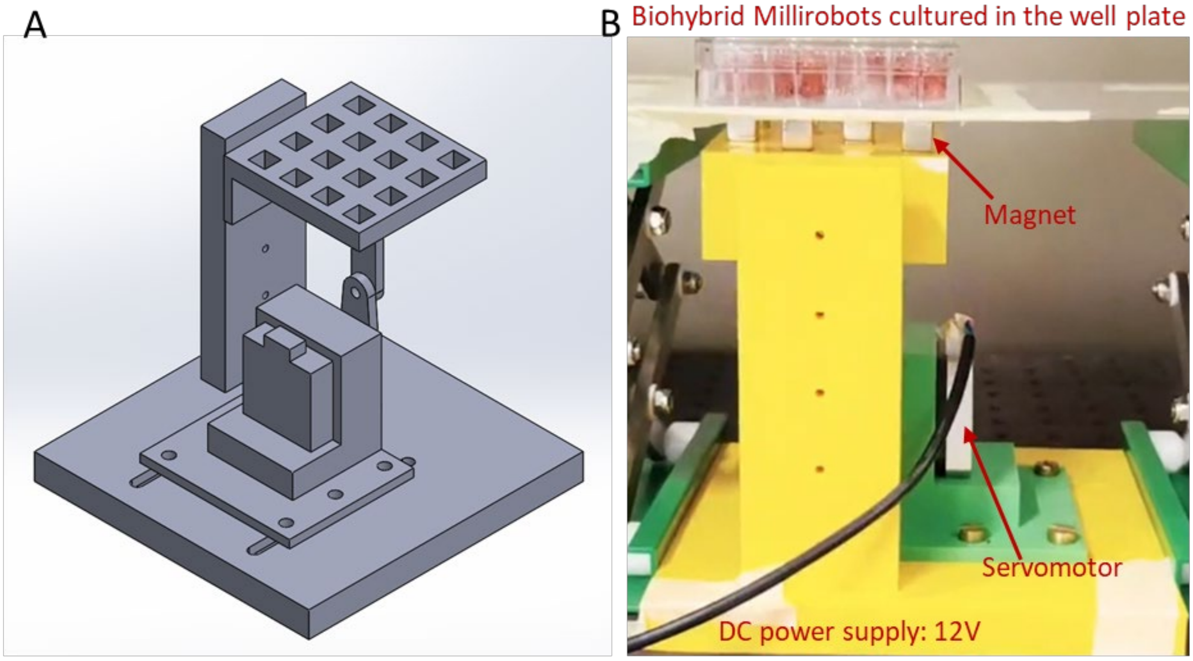

Figure S6. Magnet training device capable of reciprocating motion. (A) STL assembly setup, and (B) experimental setup for soft millirobot training, where the millirobots were cultured in a well plate positioned above the magnetic platform.

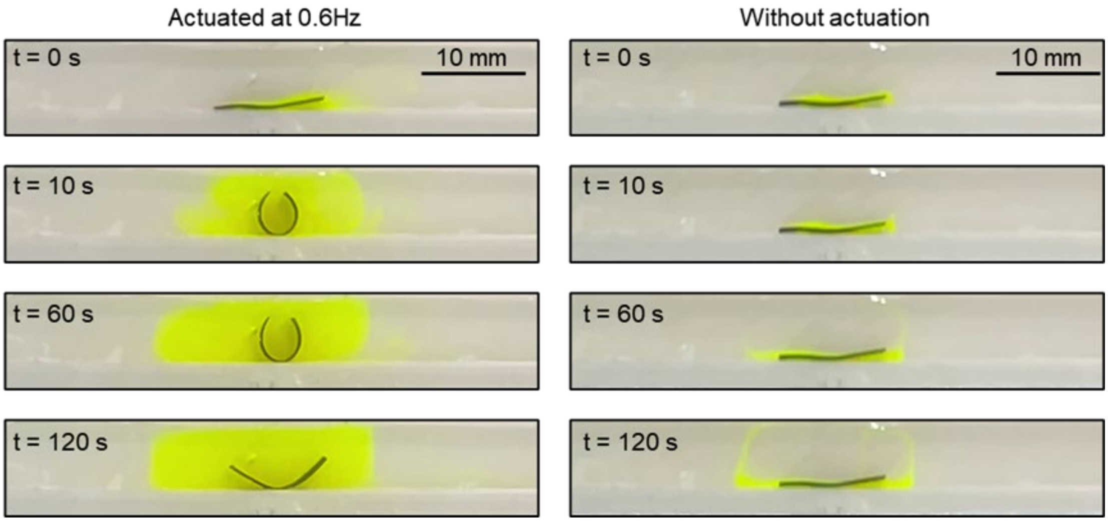

Figure S7. Comparison of dye distribution with and without millirobot actuation.

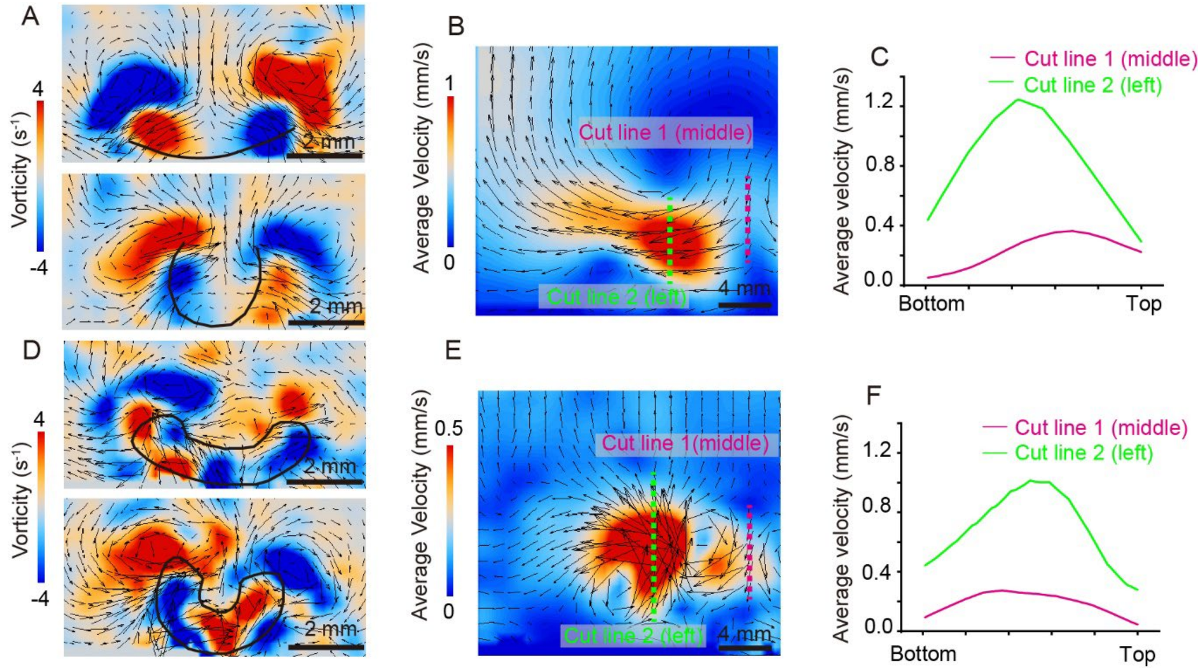

Figure S8. (A-C) Fluid transport in the 2D cell-sheet biohybrid millirobot at the highest contraction and relaxation moments (A), the average velocity map (B) and the average velocity along the cut lines (C), and (D-F) fluid transport of 3D cell-laden hydrogel millirobot at the highest contraction and relaxation moments (D), the average velocity map (E) and the average velocity along the cut lines (F).

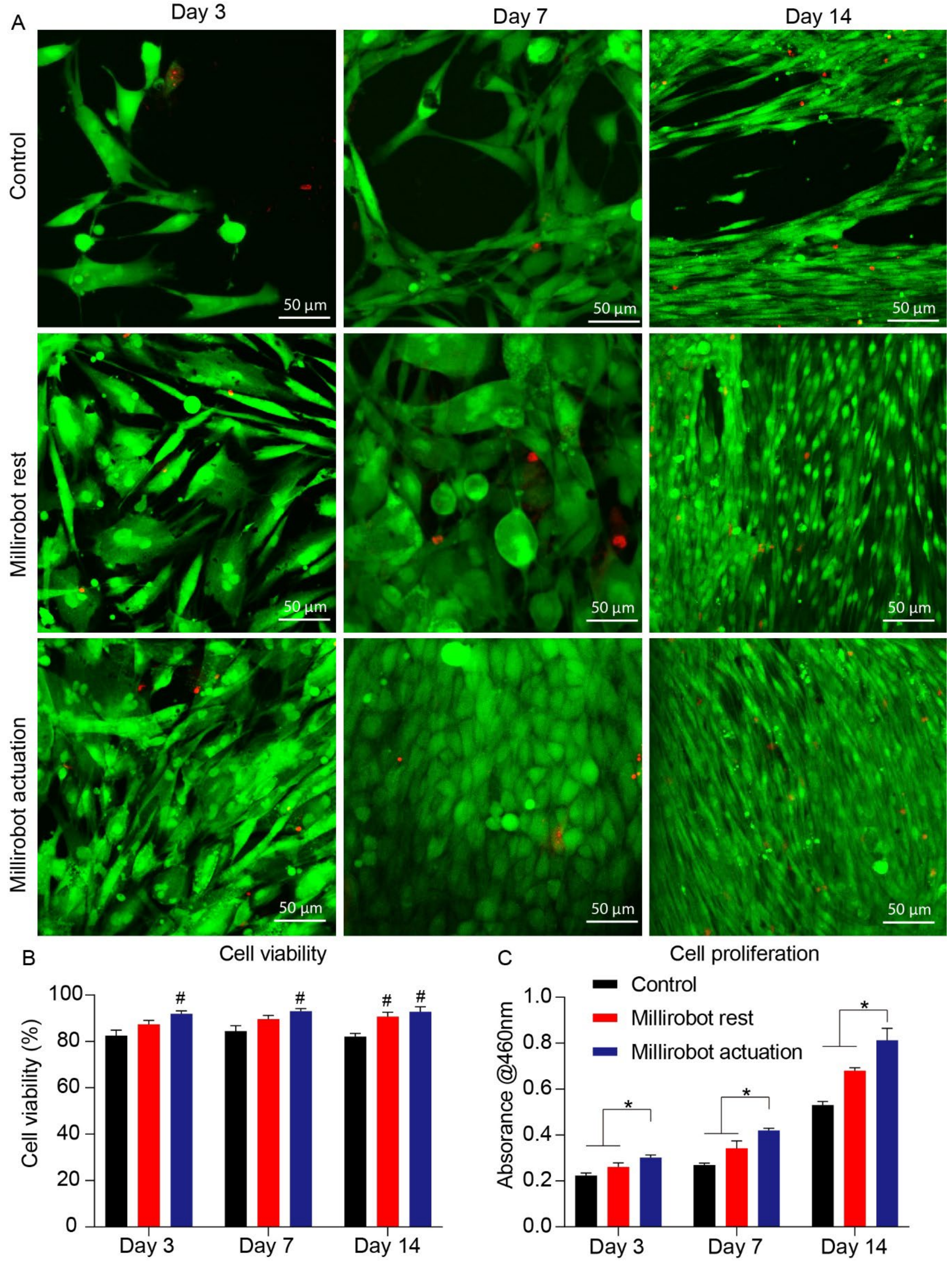

Figure S9. Biocompatibility of the 2D cell-sheet soft perforated millirobot and effects of in-situ mechano-stimulation on the hMSCs viability and proliferation. (A) Live/dead cells staining images and (B)

quantification of cell viability of hMSCs on 2D cell-sheet biohybrid millirobots under control (millirobot without fibronectin modification and rest), millirobot rest, and millirobot actuation conditions on days 3, 7, and 14. (C) Cell proliferation of hMSCs under the different conditions. * $P < 0.05$, # $P < 0.05$ compared to the control group.

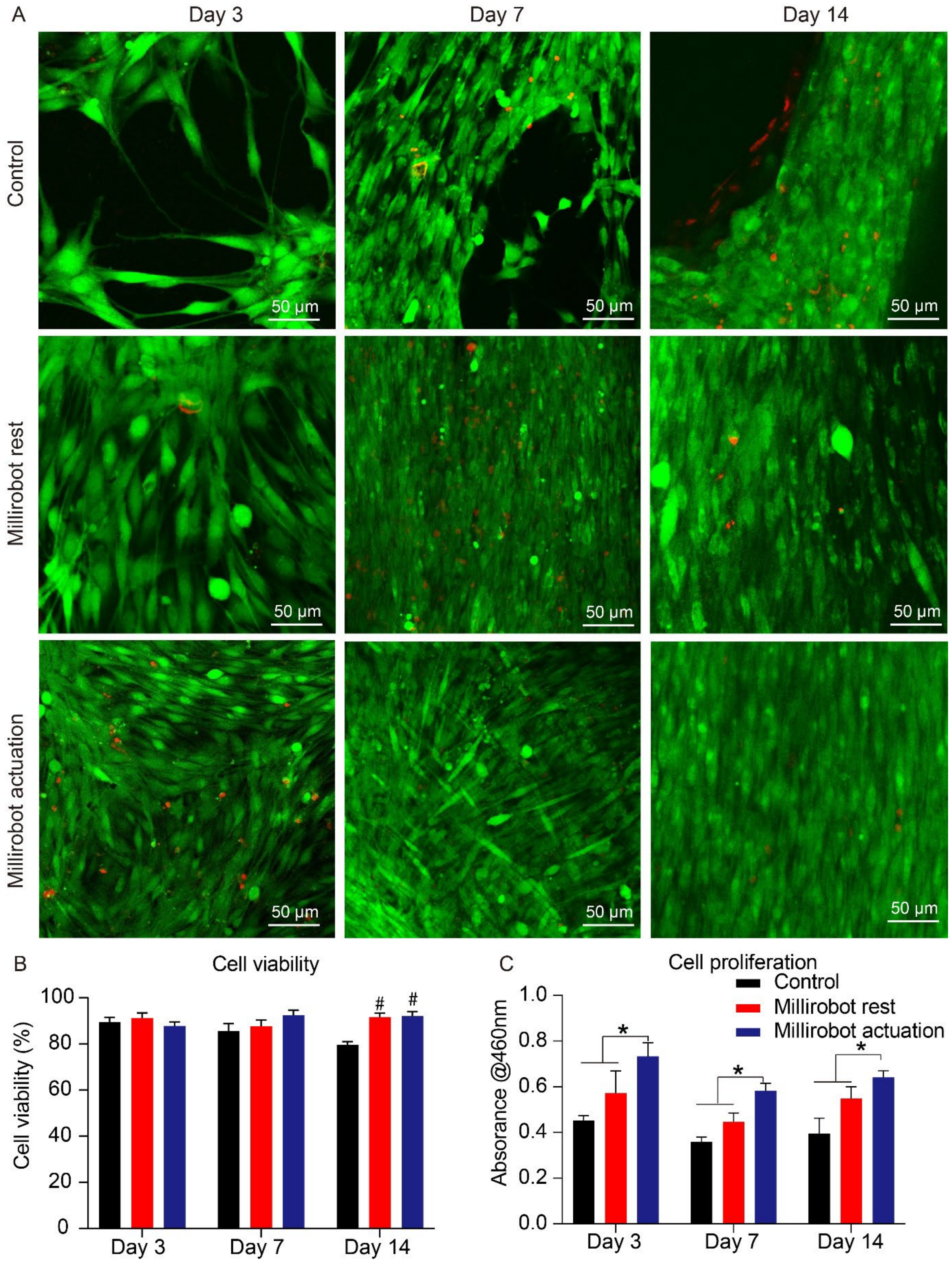

Figure S10. Biocompatibility of the 2D cell-sheet soft perforated millirobot and effects of in-situ mechano-stimulation on the 3T3 fibroblast proliferation. Live/dead staining images (A) and quantification of cell

viability (B) of 3T3 fibroblast on 2D cell-sheet biohybrid millirobots under control (millirobot without fibronectin modification and rest), millirobot rest, and millirobot actuation conditions on days 3, 7, and 14. (C) Cell proliferation of NIH-3T3 fibroblast under the different conditions. * $P < 0.05$, # $P < 0.05$ compared to the control group.

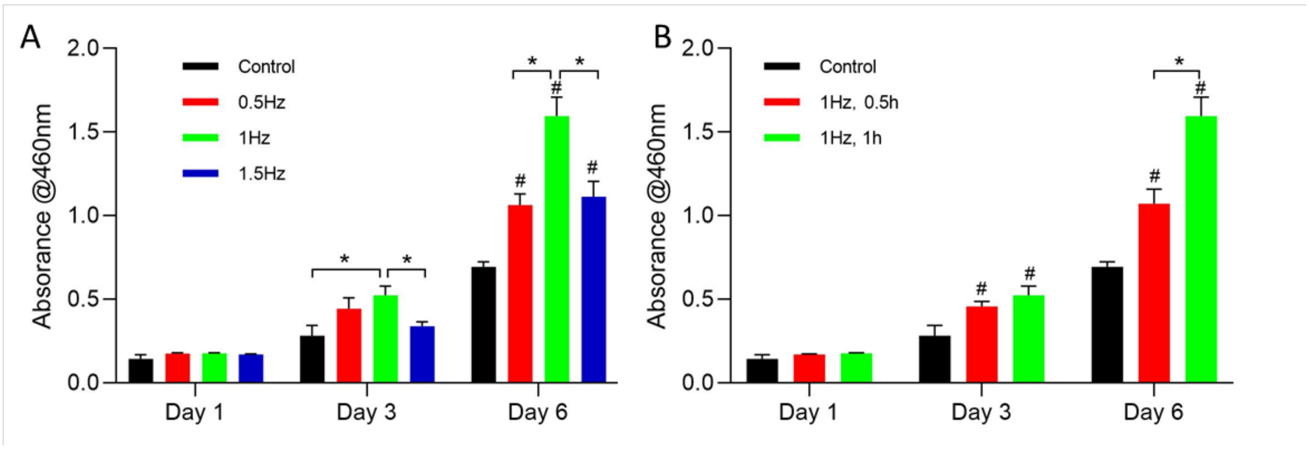

Figure S11. The influence of alternative parameters of in situ mechanical stimulation on C1C12 cell proliferation in adhesive wellplates. (A) Frequencies (control, 0.5 Hz, 1 Hz, 1.5 Hz) at 1h/day, (B) durations (control, 0.5 h, 1 h) at 1Hz frequency. * $P < 0.05$, # $P < 0.05$ compared to the control group.

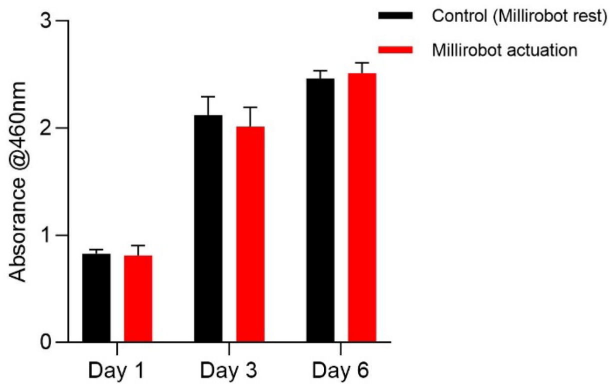

Figure S12. Cell proliferation of C2C12 cells cultured on the well plate (not on the millirobot surface). (Black) Control group: cells cultured on the well plate with the millirobot at rest. (Red) Experimental group: cells cultured on the well plate with the millirobot actuated at 1 Hz for 1 hour per day.

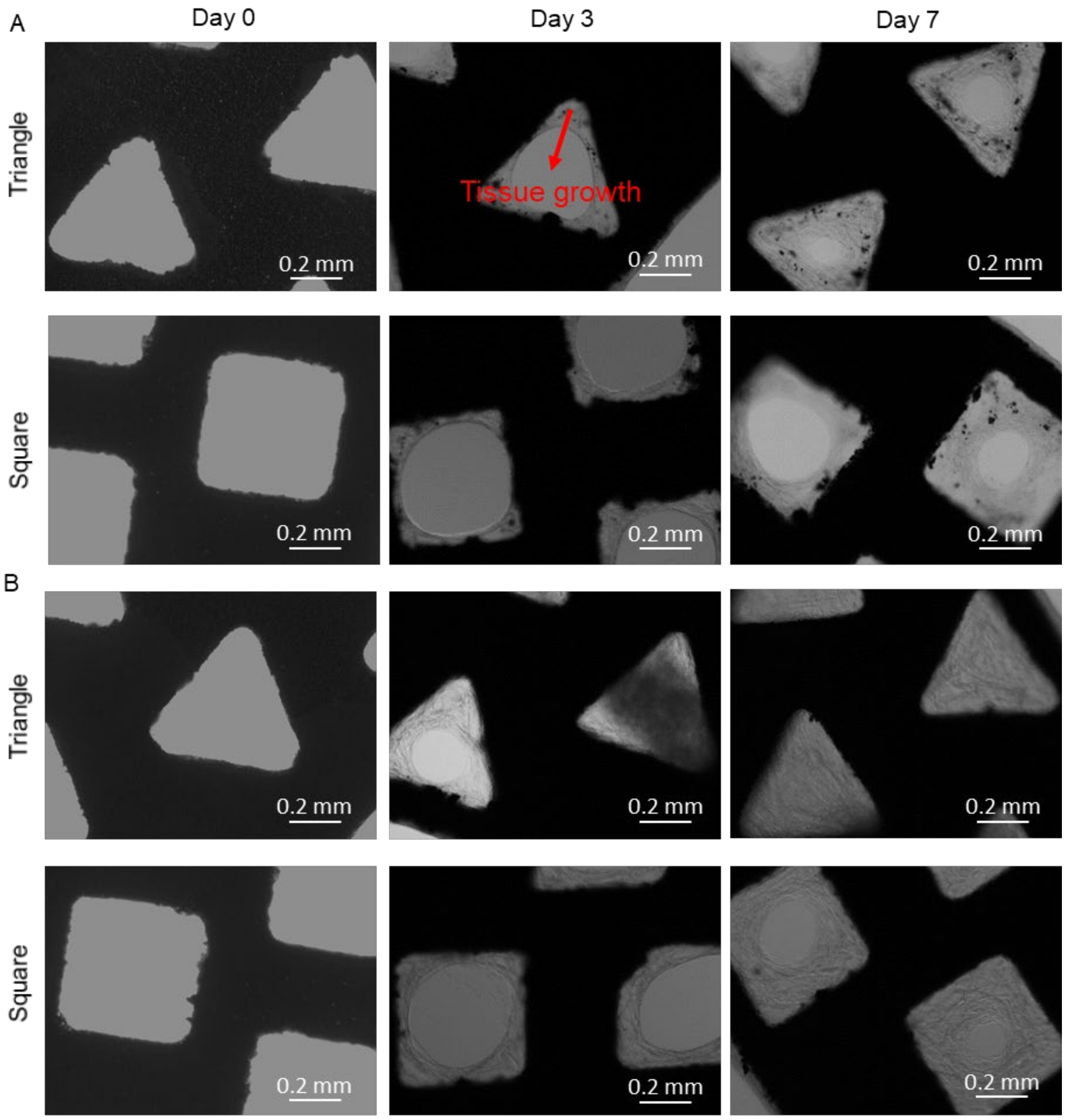

Figure S13. Cell-sheet growth of C2C12 cells on 2D soft perforated millirobots with different pore structures on day 0, 3 and 7 under millirobot rest (A) and millirobot actuation (B) conditions.

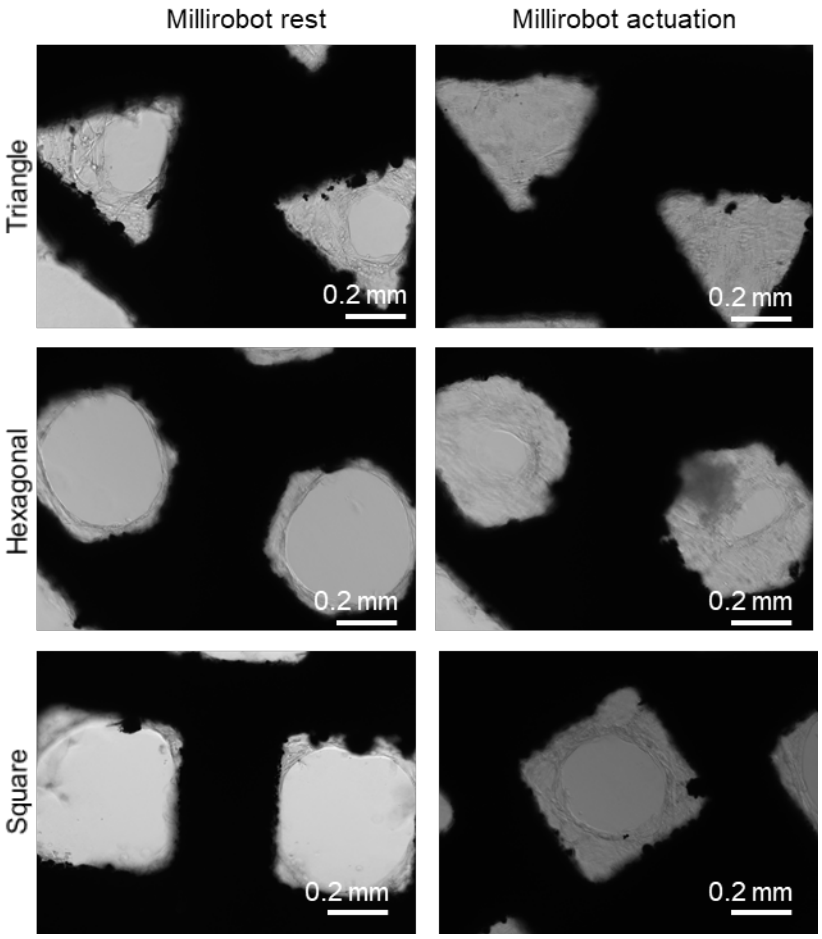

Figure S14. Cell-sheet growth of hMSCs on 2D soft perforated millirobots with different pore structures on day 3 under millirobot rest and millirobot actuation conditions.

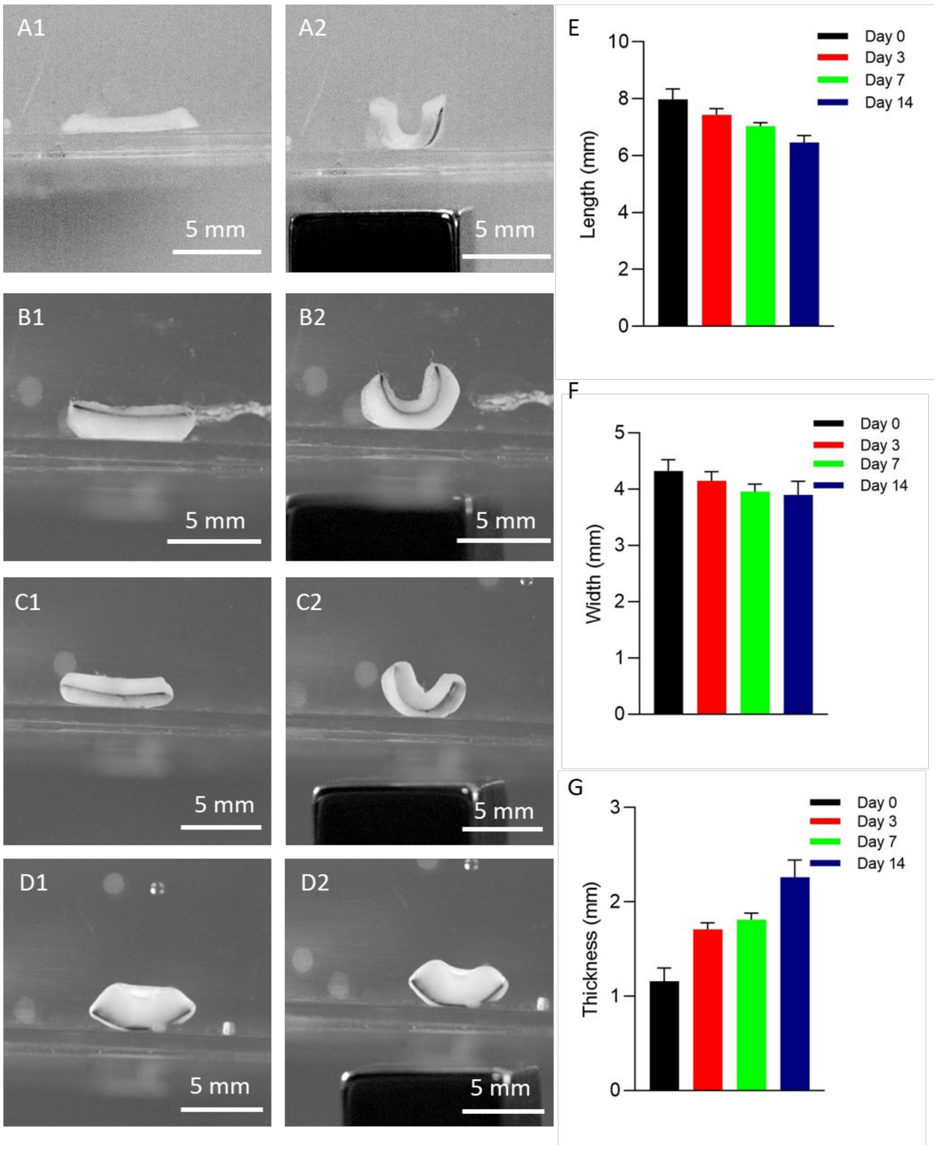

Figure S15. Temporal changes in the sample size of 3D cell-laden hydrogel millirobots. (A-D) Bright-field images of 3D cell-laden hydrogel millirobots at their maximal relaxation (A1-D1) and contraction (A2-D2) under magnetic actuation on day 0 (A1, A2), day 3 (B1, B2), day 7 (C1, C2), and day 14 (D1, D2). Quantitative analysis of changes in length (E), width (F), and thickness (G) of the 3D cell-laden hydrogel millirobots over time.

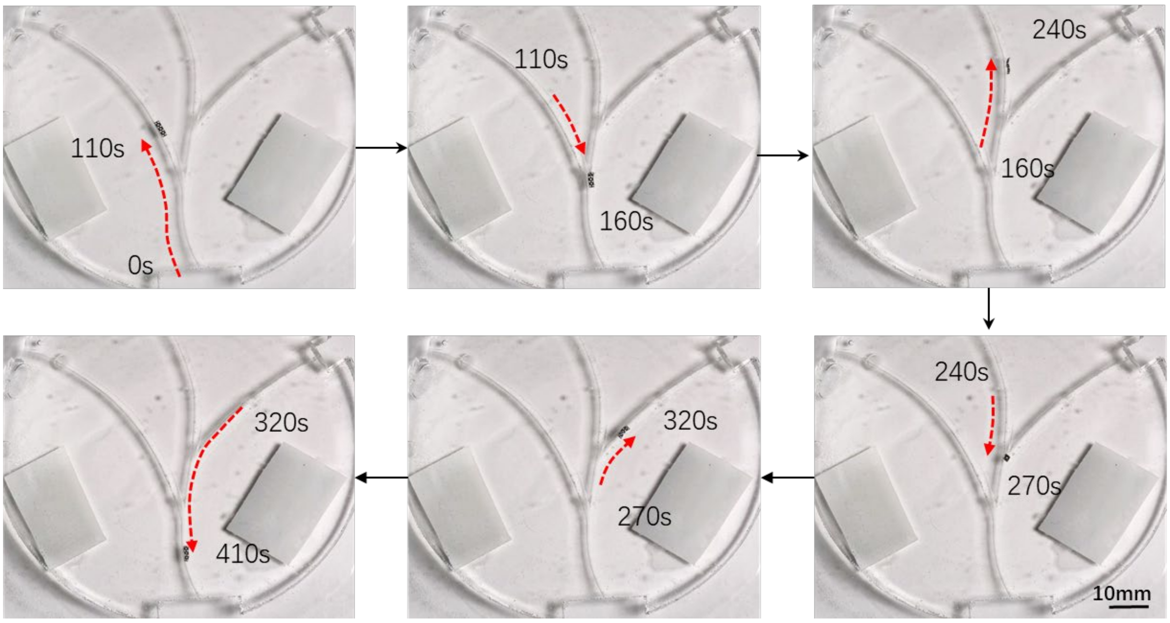

Figure S16. The locomotion of 2D cell-sheet biohybrid millirobot in the *in vitro* curved PDMS duct model.

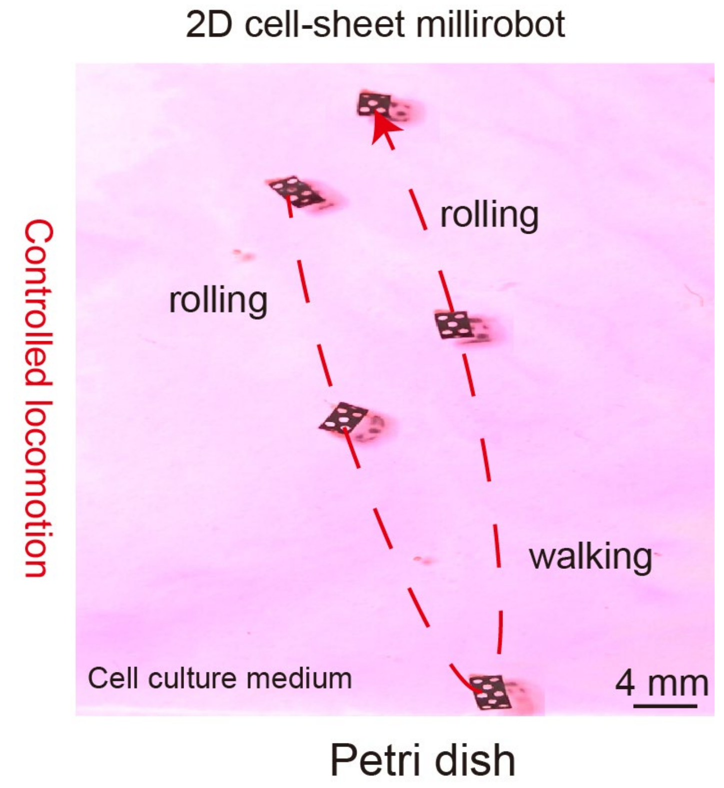

Figure S17. Controlled locomotion of a 2D cell-sheet biohybrid millirobot on a petri dish with cell culture medium via magnetic actuation *in vitro*.

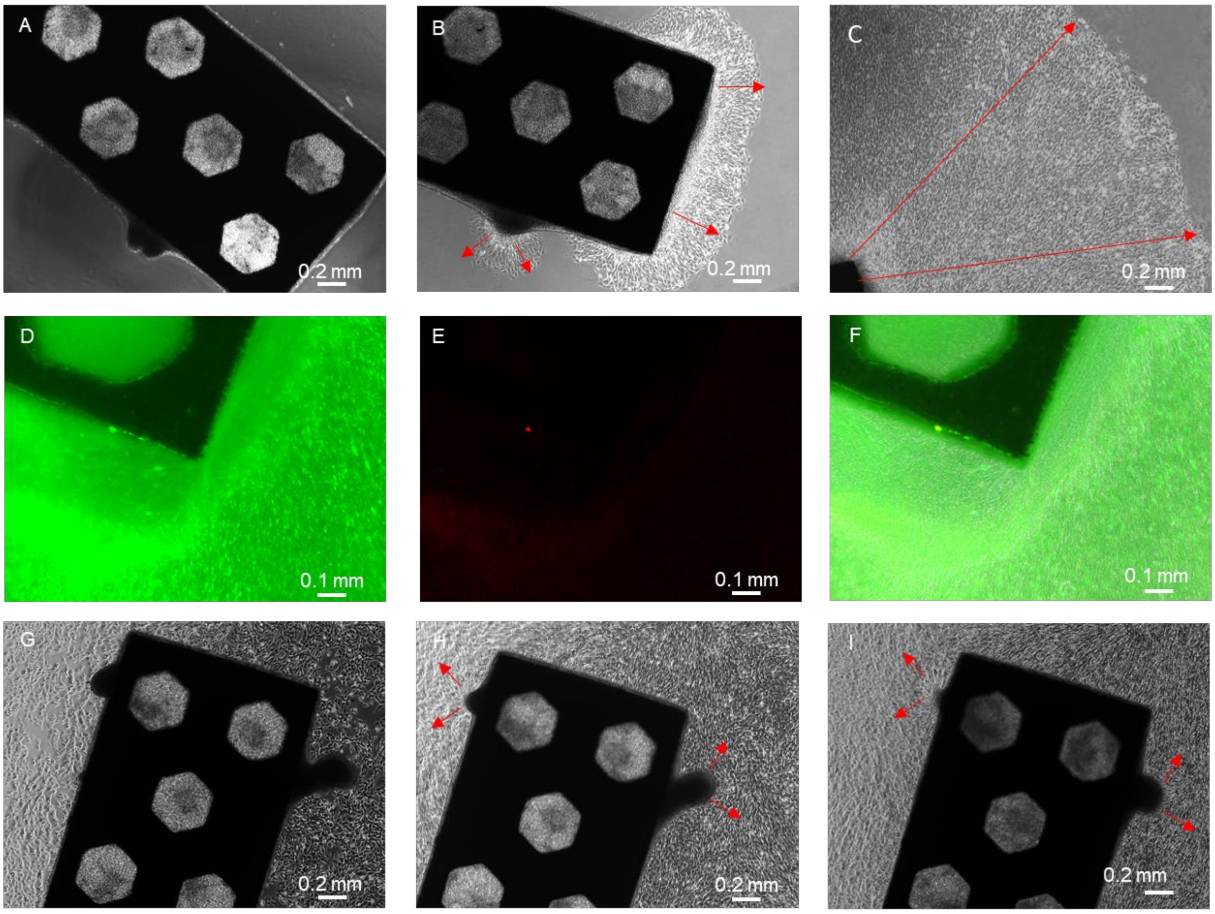

Figure S18. C2C12 cell migration from 2D cell-sheet biohybrid millirobot on the wellplate (A-C) and biofilm (G-I) at day 0 (seeding, A, G), day 1 (B, H), and day 4 (C, I). The live/dead cell staining images of cell migration on day 4, live cell image (D), dead cell image (E), merged image of bright field, live/dead staining images (F).

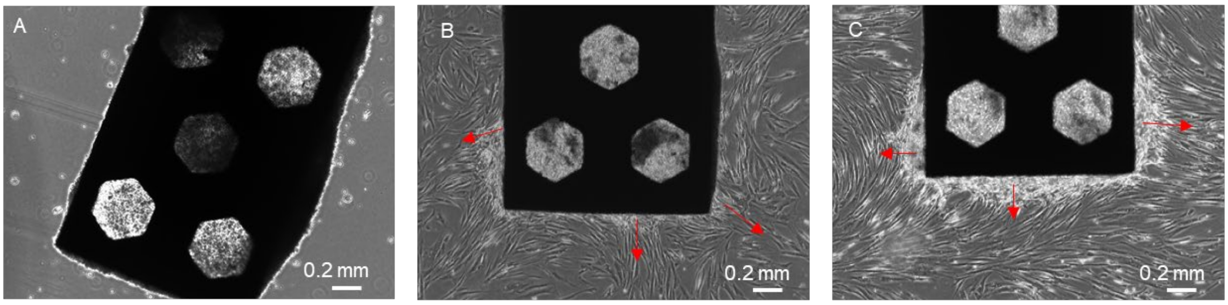

Figure S19. hMSCs cell migration from 2D cell-sheet biohybrid millirobot on the wellplate (A-C) at day 0 (seeding, A), day 4 (B), and day 9 (C).

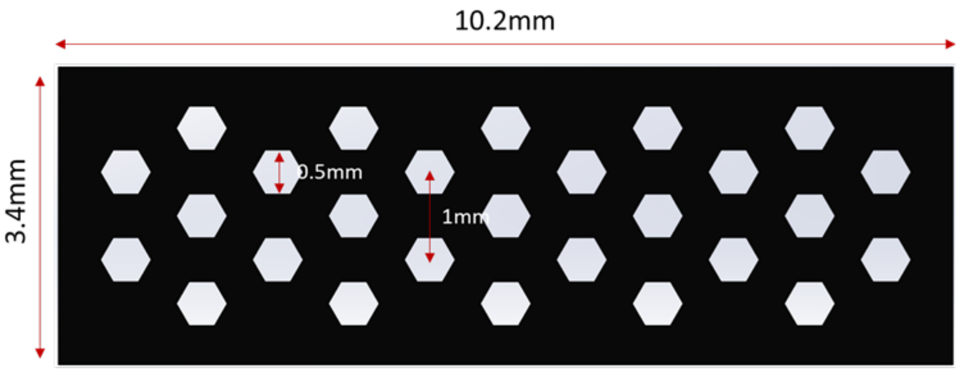

Length:	10.2mm	Pore type:	hexagon
Width:	3.4mm	Pore height:	0.5mm
Thickness:	1mm	Pore distance:	1mm

Figure S20. Sample size of millirobot for *ex vivo* muscle tissue deformation.

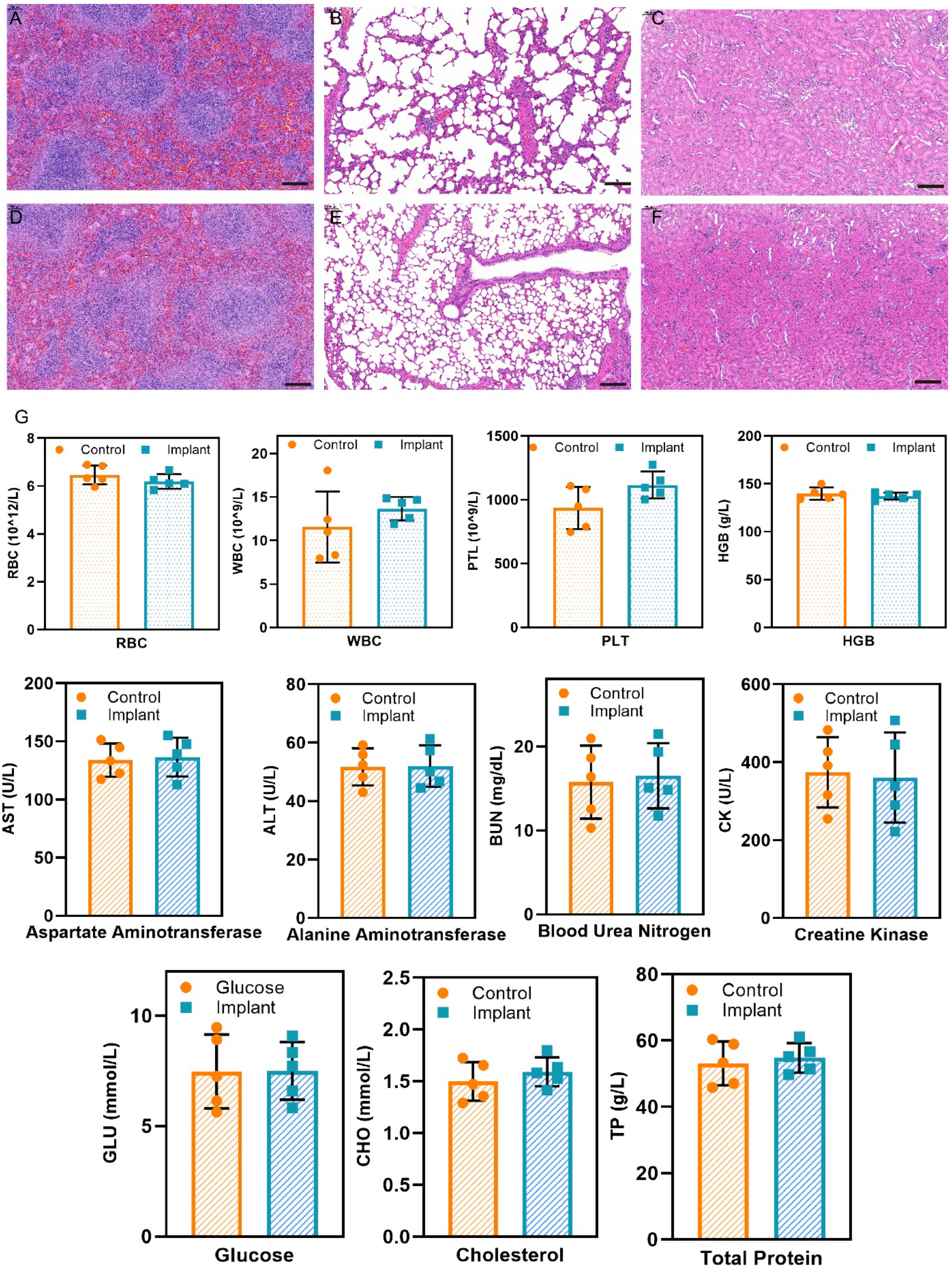

Figure S21. H&E staining of major organs (the spleen (A, D), lung (B, E), kindney (C, F)) after implantation for 14 days, (A-C) millirobot implantation, (E-F) control group, scaler bar 200 μm. (G) Hematological examination of the variation in, white blood cell count (RBC), red blood cell count (WBC), platelet count (PLT), and hemoglobin (HGB) after subcutaneous implantation for 14 days. n = 3. Blood biochemistry

examination of the variation in aspartate aminotransferase (AST) and alanine aminotransferase (ALT) after subcutaneous implantation for 14 days. n = 3. Blood biochemistry examination of the variation in blood urea nitrogen (BUN), creatine kinase (CK), blood glucose (GLU), total cholesterol (CHO), and, total protein (TP) after subcutaneous implantation for 14 days. n = 3. All data above are presented as means ± SD.

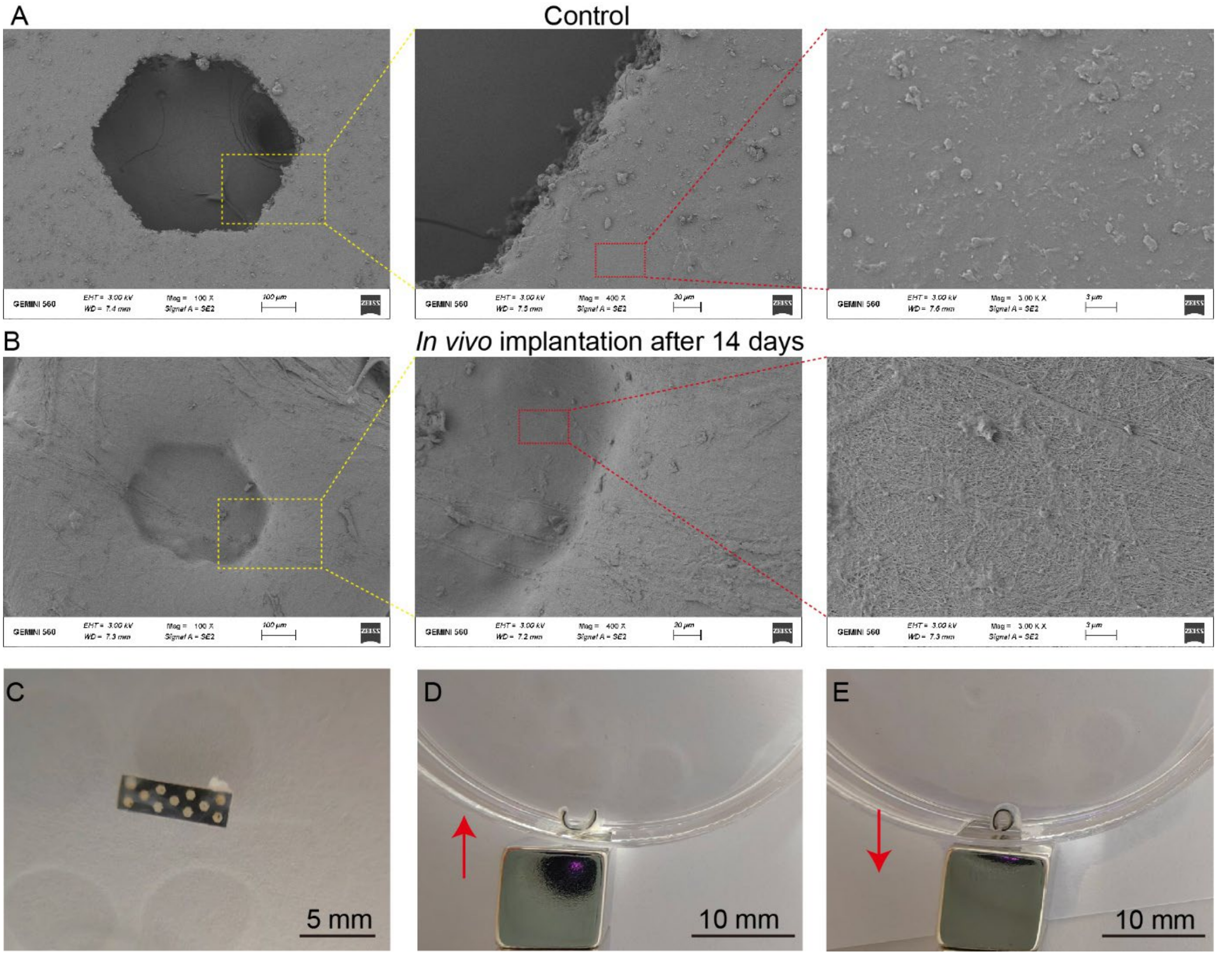

Figure S22. SEM images of 2D soft perforated millirobot before (A) and after (B) *in vivo* implantation for 14 days. (C) Tissue-millirobot integration image after *in vivo* implantation for 14 days, (D) (E) Magnetic actuation testing of the tissue–millirobot biohybrid under opposing magnetic fields.

Supplementary Videos:

Video S1. Experimental setup for soft millirobot training.

Video S2. Magnet actuation of 2D cell-sheet biohybrid millirobots on day 0 and day 7.

Video S3. Electrical stimulation of 2D cell-sheet biohybrid millirobots with millirobot rest and millirobot actuation conditions on day 28.

Video S4. Myotube contraction distance tracking in the 2D cell-sheet biohybrid millirobot with in-situ mechano-stimulation on day 28.

Video S5. Magnet actuation of 3D cell-laden hydrogel millirobots on day 0 and day 7.

Video S6. 2D cell-sheet biohybrid millirobot locomotion in *ex vivo* pig liver model under magnetic actuation.

Video S7. 3D cell-laden hydrogel millirobot locomotion in *ex vivo* pig liver model under magnetic actuation.

Video S8. 2D cell-sheet biohybrid millirobot locomotion in *ex vivo* pig liver model under magnetic actuation after target delivery.

Video S9. 3D cell-laden hydrogel millirobot locomotion in *ex vivo* pig liver model under magnetic actuation after target delivery.

Video S10. Ex vivo locomotion of 2D cell-sheet biohybrid millirobot in a complex curved duct liver model

Video S11. *In vitro* locomotion of 2D cell-sheet biohybrid millirobot in a complex curved duct model

Video S12. *In-vitro* controlled locomotion of 2D cell-sheet biohybrid millirobot.

Video S13. *In-vitro* controlled locomotion of 3D cell-laden biohybrid millirobot.

Video S14. 2D cell-sheet millirobot locomotion and on-site mechano-stimulation on an ex vivo muscle tissue model

Video S15. 3D cell-laden millirobot locomotion and on-site mechano-stimulation on an ex vivo muscle tissue model

Video S16. Effects of 2D soft millirobot actuation on *ex vivo* muscle tissue deformation under magnet actuation.

Video S17. Integration of the tissue-millirobot interface following 14 days of implantation
